# Fully Biodegradable Packaging Films for Fresh Food Storage Based on Oil‐Infused Bacterial Cellulose

**DOI:** 10.1002/advs.202400826

**Published:** 2024-04-03

**Authors:** Guoli Chen, Kaimin Wang, Pinghang Chen, Daohang Cai, Yan Shao, Rui Xia, Chun Li, Haochuan Wang, Fuzeng Ren, Xing Cheng, Yanhao Yu

**Affiliations:** ^1^ Department of Materials Science and Engineering Southern University of Science and Technology Shenzhen 518055 China; ^2^ Institute of Innovative Materials Southern University of Science and Technology Shenzhen 518055 China

**Keywords:** bacterial cellulose, biodegradable packaging film, fresh food storage, oil infusion

## Abstract

Fully biodegradable packaging materials are demanded to resolve the issue of plastic pollution. However, the fresh food storage performance of biodegradable materials is generally much lower than that of plastics due to their high permeability, microbial friendliness, and limited stretchability and transparency. Here a biodegradable packaging material is reported with high fresh food storage performance based on an oil‐infused bacterial cellulose (OBC) porous film. The oil infusion significantly improved cellulose's food‐keeping performance by reducing its gas permeability, increasing its stretchability and transparency, and enabling the active release of green vapor‐phase preservative molecules, while maintaining its intrinsically high degradability. Strawberries stored in a container with the OBC lid at 23 °C after 5 days exhibited a moldy rate of 0%, in contrast to the 100% moldy rate of those stored by poly(ethylene). Enhanced storage performance is also obtained on tomatoes, pork, and shrimp. The OBC film is naturally degraded after being buried in wet soil at 30 °C for 9 days, identical to the degradation rate of bacterial cellulose. The liquid seal strategy broadly applies to different celluloses, providing a general option for developing cellulose‐based biodegradable packaging materials.

## Graphical abstract

A biodegradable packaging material with changeable properties is developed by combining a biocompatible oil with an intrinsically degradable bacterial cellulose film. The dynamic oil alters cellulose's optical, mechanical, barrier, and antimicrobial properties in the favorable direction of fresh food storage, leading to improved food‐keeping performance compared with commercial polyethylene plastics while being quickly degradable.

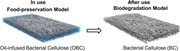

## 1 Introduction

Food packaging annually consumes over 100 million tons of petroleum‐based plastics such as poly(ethylene) (PE) and poly(ethylene terephthalate) (PET) all over the world, causing severe environmental pollution.^[^ ^1^, ^2^, ^3^, ^4^, ^5^ ^]^ Biodegradable packaging materials are promising solutions.^[^ ^6^ ^]^ For example, cellulose straws avoided the use of 500 million plastic straws per day in the United States.^[^ ^7^ ^]^ Applying cellulose to a broader range of food packaging is hindered by the conflict of material properties needed for food storage and biodegradability.^[^ ^8^ ^]^ The high degradation rate of cellulose (typically degradable in natural soil within one month) originates from its hydrophilic nature and porous structure, which allow microorganisms and enzymes to attach and function.^[^ ^9^, ^10^, ^11^, ^12^, ^13^ ^]^ However, these characteristics also make the cellulose film gas‐permeable,^[^ ^8^ ^]^ non‐transparent,^[^ ^14^ ^]^ and microbial‐friendly,^[^ ^15^ ^]^ causing water loss, inconvenience of use, and spoilage of stored fresh food.

Strategies to improve cellulose's packaging performance include coating a thin layer of PE plastic onto the cellulose film to increase its mechanical and barrier properties,^[^ ^16^ ^]^ chemically treating cellulose fibers with hydrophobic molecules to reduce water permeability,^[^ ^17^, ^18^ ^]^ and incorporating antimicrobial particles or emulsions to prevent food spoilage.^[^ ^19^, ^20^, ^21^ ^]^ Nevertheless, plastic integration and hydrophobic treatment substantially decreased the degradation rate of cellulose.^[^ ^16^, ^18^ ^]^ The antimicrobial particles (e.g., Ag and TiO_2_) had to be in direct contact with food, resulting in a limited functioning area.^[^ ^22^, ^23^, ^24^ ^]^ The emulsions (e.g., essential oil in water) provided a favorable vapor‐phase antimicrobial environment by evaporation, but largely increased the opacity of the cellulose film.^[^ ^25^, ^26^ ^]^ A design that comprehensively improves cellulose's packaging performance without affecting its biodegradability remains inaccessible.

Oil infusion is a versatile strategy that can adjust the optical,^[^ ^27^ ^]^ mechanical,^[^ ^28^ ^]^ and transport^[^ ^29^ ^]^ properties of porous solids. The liquid content can also serve as a resourceful reservoir that carries chemical species to endow the solid with otherwise unreachable biological functions.^[^ ^30^ ^]^ The intimacy between oil and solid is dynamically tunable to make the oil stably held in the solid during operation, while being fully removable upon chemical stimuli after usage.^[^ ^31^, ^32^ ^]^ These characteristics align well with the requirements of cellulose‐based packaging materials.

**Figure 1:**
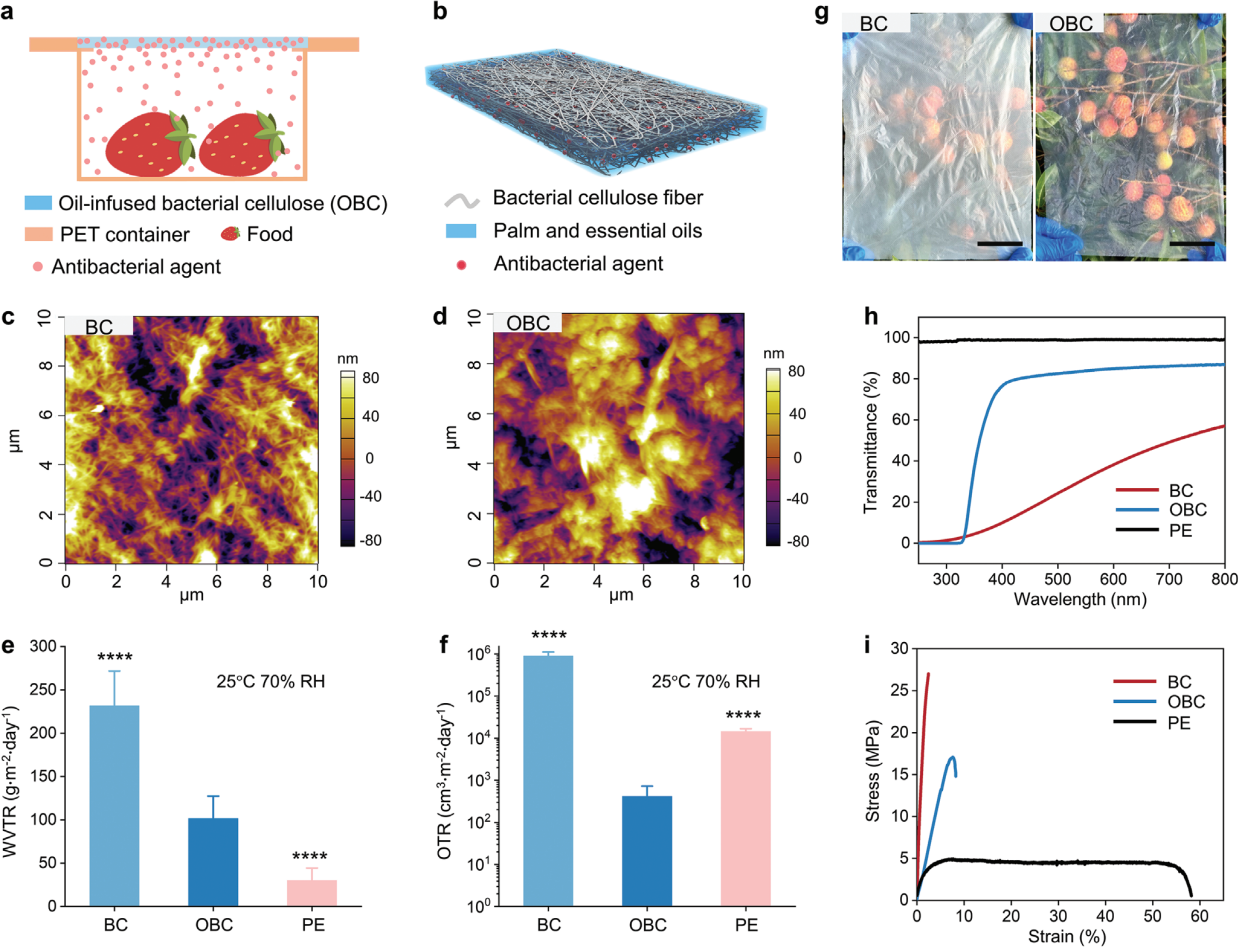
Structure and property of OBC. a) Schematic illustration of using the OBC film as a packaging lid for storing fresh food. b) Schematic showing the structure of the OBC film. c,d) AFM topographic images of the BC (c) and OBC (d) films. e,f) Comparisons of water vapor transmission rates (e) and oxygen transmission rates (f) of BC, OBC, and commercial PE films with the same film thickness of 15 µm. ^∗∗∗∗^ *p* < 0.0001 by one‐way ANOVA, compared with the response of the OBC group. g) Photographs of BC and OBC films in front of lychee. Scale bars are 5 cm. h) Transmittance measurement at UV and visible light regions for BC, OBC, and PE films. i) Stress–strain curves of BC, OBC, and PE films.

Here, we developed a naturally degradable packaging material with high food‐keeping performance based on amphiphilic porous bacterial cellulose (BC) film infused by palm and essential oils doped with antimicrobial agents, so‐called oil‐infused bacterial cellulose (OBC). The OBC film was obtained by immersing a porous BC film in the oil bath, through which the oil was stably locked in the pores by capillary force due to the lipophilic feature of BC. Oil infusion dramatically altered BC's physiochemical properties, including reducing water and oxygen vapor transmission rates (WVTR and OTR), extending stretchability, increasing transparency of visible light, decreasing transmittance of ultraviolet light, and adding antimicrobial function, with the only exception of high biodegradability. Strawberries stored in a container capped with the OBC film remained in the freshest condition in terms of moldy rate, nutrient content, weight loss, and firmness, compared with those stored under a BC lid, a PE lid, or no lid condition (**Figure** **1**a).

## 2 Results and Discussion

We chose porous BC film as the solid substrate considering its amphiphilic nature that is necessitated for oil wetting (Figure 1b). Palm and essential oils were selected as the main content since these are natural products with high biosafety and have a basic antimicrobial function.^[^ ^33^ ^]^ The essential oils are thyme essential, mainly composed of thymol (content >40 wt.%) and caryophyllene (Figure S1, Supporting Information). Cinnamaldehyde molecules (also a natural product) were introduced into the oil as dopants to further enhance its antimicrobial properties. We chose to combine essential oils with cinnamaldehyde, rather than directly using cinnamon essential oil, for precisely controlling the content of cinnamaldehyde in essential oils during the research. Otherwise, cinnamon essential oil is a more reasonable choice.

The oil and water contact angles of BC were 34.1° and 35.2°, respectively, verifying its amphiphilic characteristic (Figure S2, Supporting Information). The BC film has a fabric porous structure with a fiber diameter of 10–200 nm, a pore diameter of 10–100 nm, and a type I crystal structure (Figure S3, Supporting Information).^[^ ^34^ ^]^ As a consequence of the lipophilic property and porous structure of BC, a stable liquid‐solid composite (i.e., the OBC film) was obtained after immersing the BC film in a mixture of palm oil, essential oil, and cinnamaldehyde (volume ratio of 5:5:1). The oil composition can be broadly tunable from pure palm oil to cinnamaldehyde‐rich oils, depending on the application scenarios. The mass ratio between the liquid and solid contents was controlled between 39% and 52%. The cinnamaldehyde molecules can evaporate and provide a gas‐phase antimicrobial environment and a non‐irritating odor of cinnamon, while the palm oil was thermally stable in OBC (Figure S4, Supporting Information). The OBC film has a thin and air‐stable oil layer on the surface, similar to other oil‐infused porous materials.^[^ ^35^, ^36^ ^]^ The chemical properties and microstructure of the film were stable in the air (Figures S4 and S5, Supporting Information). Atomic force microscopy (AFM) confirmed the porous structure of BC film and showed a slightly larger pore diameter compared with that observed by scanning electron microscopy (SEM, Figure S3a, Supporting Information), because of the lower field depth of AFM (Figure 1c). The fiber and pore features were vaguer in the OBC film due to the oil infusion (Figure 1d). 3D fluorescence microscopy revealed that the oil was uniformly distributed inside the BC film (Figure S6, Supporting Information).

A 15 µm‐thick OBC film has a WVTR of 101.7 g m^−2^ day^−1^ at 25 °C and 70% relative humidity (RH), 56% lower than that of a BC film with the same thickness (231.5 g m^−2^ day^−1^, Figure 1e). PE has the lowest WVTR of 30.1 g m^−2^ day^−1^ due to its hydrophobic nature. The OTR of OBC, PE, and BC were 418, 14530, and 901220 cm^3^ m^−2^ day^−1^, respectively (Figure 1f). The orders‐of‐magnitude improvement in the oxygen barrier performance for OBC was a combined result of the physical repellency and chemical consumption of oxygen by the infused oil.^[^ ^37^, ^38^ ^]^ The low gas permeability is favorable for preventing food from water loss and oxidation‐induced spoilage.

The OBC film is transparent for visible light and opaque for UV light (Figure 1g,h). The transmittance of OBC at the wavelength of 500 nm reached 82.6%, 3.4 times higher than that of BC (24.3%, Figure 1h). For BC, light traveled in air and was directly scattered by the fiber at the air‐fiber interface due to the difference in refractive index (air 1 vs. cellulose 1.6),^[^ ^39^ ^]^ making it a white film. For OBC, light traveled in the oil, which has a comparable refractive index (close to 1.5) with that of cellulose fiber.^[^ ^40^ ^]^ Consequently, the light scattering effect was much weaker, making OBC transparent to visible light. The transmittance of UV light in BC and OBC was nearly zero due to the absorbance and scattering of cellulose fibers. In contrast, PE was fully transparent to UV light. Blocking UV light can prevent the formation of UV‐induced reactive oxygen species and free radicals, resulting in delayed food degradation.^[^ ^41^ ^]^ Being transparent to visible light is essential for ease of use. The optical property of OBC is therefore ideal for food storage.

The oil infusion made the BC film softer and more stretchable. The elastic modulus of the BC film was decreased from 11.1 to 2.3 MPa, the elongation at break was increased from 2.4% to 8.2%, and the tensile strength was decreased from 27.1 to 17.0 MPa (Figure 1i). These mechanical property changes originate from the weakened hydrogen bonding and van der Waals interaction between cellulose molecules due to the oil infusion.^[^ ^42^ ^]^

**Figure 2:**
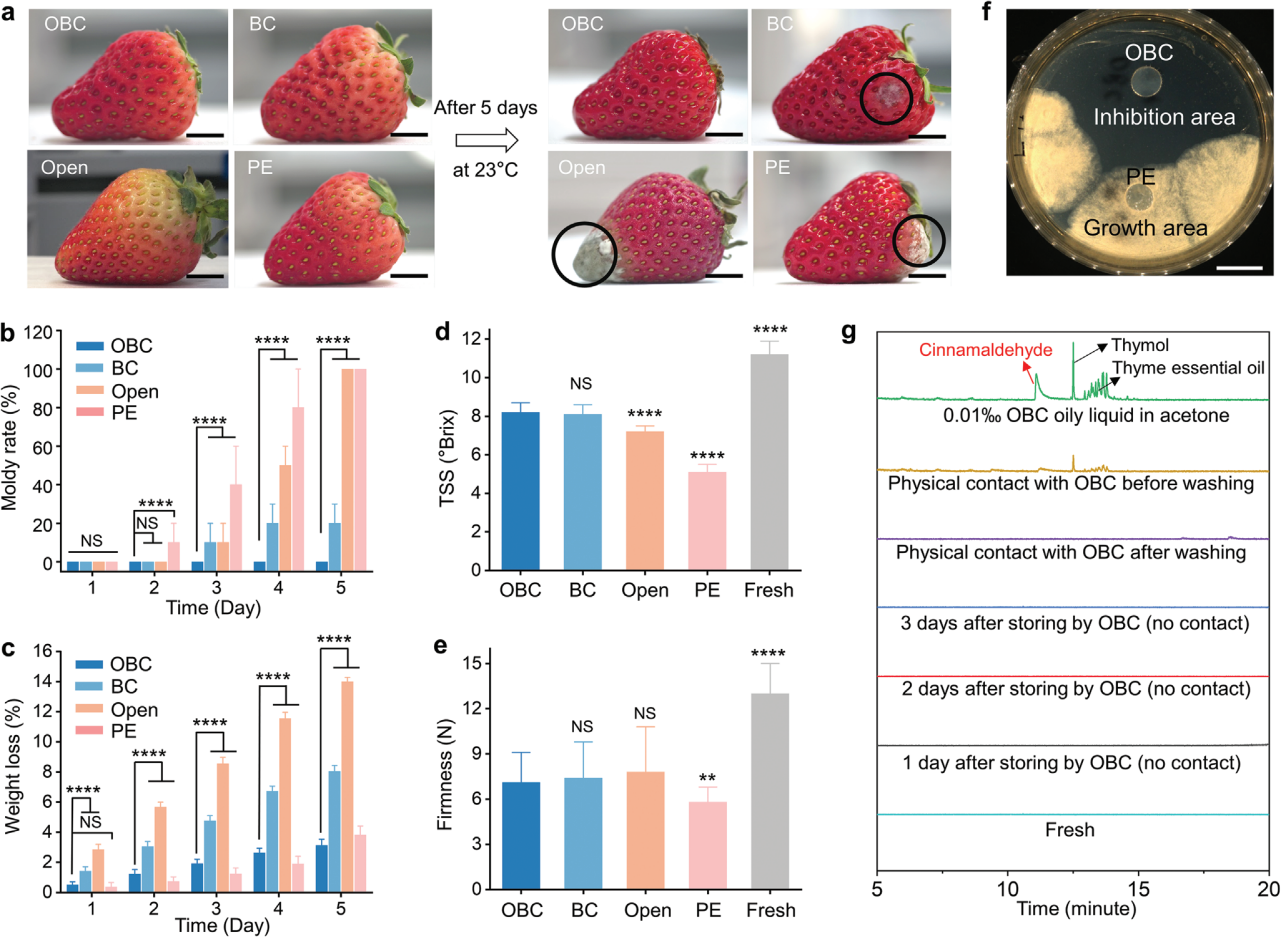
Food storage performance of OBC. a) Photographs comparing the appearance changes of strawberries before and after being stored in OBC‐sealed, BC‐sealed, PE‐sealed, and open containers at 23 °C for 5 days. The black circles point out the moldy areas. Scale bars are 1 cm. b–e), Moldy rates (b), weight loss (c), total soluble solids (TSS) contents in °Brix (d), and firmness (e) measured from 10 strawberries stored in OBC, BC, PE, and open conditions at 23 °C for 5 days. NS, not significant (*p*>0.05), ^∗∗^ *p* < 0.01, ^∗∗∗∗^ *p* < 0.0001 by one‐way ANOVA, compared with the response of the OBC group. f) Comparison of the antifungal property of OBC and PE. *Botrytis cinerea* was used as the testing microbial since it is one of the most widespread and destructive fungal diseases of horticultural crops. Scale bar is 4 cm. g) Gas chromatography for the surface substance of OBC‐packaged strawberries.

Strawberries were selected as the exemplary food being packaged considering its high sensitivity to the storage environment. As shown in **Figure** **2**a, the OBC‐packaged strawberries remained fresh after 5 days of storage at 23 °C (all food storage experiments were conducted at 23 °C unless otherwise noticed), but the PE‐ or BC‐packaged, and openly stored strawberries became moldy. Statistically, 10% of 10 strawberries in the PE group molded on day 2, and all of them molded on day 5 (Figure 2b). The PE has a low WVTR and thus confines the moisture generated by the transpiration of strawberries inside the container, leading to a humid environment that is favorable for internal microbial reproduction and mold generation.^[^ ^43^ ^]^ For a different reason, strawberries placed in the open condition also had a 100% mold rate. The open condition made the strawberries directly exposed to the air and continuously subjected to external microorganisms that could produce mold. For the BC film, 20% of strawberries became moldy on day 5. The lower moldy rate compared to PE and open was a result of BC's high WVTR and isolation of external microorganisms. The OBC film delivered a 0% moldy rate after 5 days regardless of its relatively low WVTR, significantly outperforming the porous poly(lactic acid) (PLA) film loaded with thyme essential oil, which had a moldy rate of 37%.^[^ ^44^ ^]^ The high storage performance in the humid condition was attributed to the gradually released anti‐fungal molecules, including cinnamaldehyde and thymol, from the liquid oil in OBC to the gas environment in the container (Figure S7, Supporting Information).

Aside from mold, water loss caused by transpiration is another issue that deteriorates the freshness of fruits. The weight loss of strawberries stored in the open and BC‐packaged conditions reached a high level of 14.0% and 8.0% after 5 days, respectively, due to the fast air exchange surrounding the strawberries (Figure 2c). In contrast, the weight loss of strawberries stored in OBC‐ and PE‐packaged conditions were maintained at a low level of 3.1% and 3.8% after 5 days (statistically significantly different), respectively, as a consequence of their low WVTR. The sudden increase in weight loss from day 4 to day 5 for the PE group was caused by the juice flow after the over‐ripening of strawberries (Figure S8, Supporting Information). The oxygen content inside the OBC‐sealed container remained at above 10% after 5 days (Figure S9, Supporting Information). This oxygen content was higher than that of commercial storage conditions for strawberries (5–10%) and above the threshold for anaerobic respiration (3%).^[^ ^45^, ^46^ ^]^

The appropriate WVTR value depends on the type of packaging film and the category of the packaged food. For a non‐antimicrobial film, too low or too high WVTR both induce food spoilage. Low WVTR causes food spoilage by producing mold (Figure 2b, PE condition), while high WVTR causes food spoilage through water loss (Figure 2c, open and BC conditions). In general, the appropriate WVTR of a non‐antimicrobial film for fruits and vegetables ranges from 10 to 3000 g m^−2^ day^−1^ and that for meats ranges from 10 to 60 g m^−2^ day^−1^.^[^ ^47^ ^]^ However, if the film was antimicrobial, the WVTR should be as low as possible since the antimicrobial agents can suppress the food mold induced by the proliferation of microorganisms (low WVTR), but cannot avoid the spoilage induced by the water loss (high WVTR).

The value of total soluble solids (TSS) is an important parameter that indicates the nutrient content of fruits after storage. The TSS of strawberries stored under OBC, BC, PE, and open conditions after 5 days were 8.2, 8.1, 7.2, and 5.1 °Brix (the OBC group data were not significantly different from the BC group data, but were significantly different from the Open, PE, and Fresh group data), respectively, corresponding to 73.2%, 72.3%, 64.3%, and 45.5% of that in fresh strawberries (11.2, Figure 2d). The nutrient was mainly consumed by microbials that made mold. The high residual nutrient contents in the OBC and BC groups agreed with their low moldy rates observed in Figure 2b. For the PE case, strawberries lost nutrients through a combined effect of molding and over‐ripening. The over‐ripening of strawberries in the PE package was further evidenced by its significantly reduced firmness (5.8 N) compared with that of strawberries in OBC, BC, and open conditions (7.1 N, 7.4 N, and 7.8 N, respectively, Figure 2e, the OBC group data were not significantly different from the BC and Open group data, but were significantly different from the PE, and Fresh group data). The slight increase of firmness from OBC to open conditions was due to the gradual increase in water loss. The firmness of fresh strawberries reached 13 N, nearly twice higher than all samples stored after 5 days, meaning the over‐ripening effect generally existed at 23 °C.

At a refrigerator temperature of 3 °C, the strawberries stored in OBC, BC, PE, and open conditions after 5 days were all free of mold, suggesting that microbial reproduction was effectively inhibited by the low temperature (Figure S10, Supporting Information). The release rate of the antimicrobial molecules in the OBC film was temperature‐dependent and considerably slower at 3 °C compared with that at 23 °C, aligning well with the need for antimicrobial agents at room temperature, but not at refrigerator temperature (Figure S11, Supporting Information).

To disclose the functions of different contents in the oil mixture (i.e., palm oil, essential oil, and cinnamaldehyde), we compared the storing performance of palm‐oil‐infused BC and essential‐oil‐infused BC films by packaging cherry tomatoes. Cinnamaldehyde was excluded since tomatoes were less prone to spoilage compared with strawberries. After 7 days of storage at 23 °C, tomatoes placed in the open condition and the ones packaged by PE showed apparent water loss and mold effect, respectively (Figure S12, Supporting Information). Tomatoes packaged with palm‐oil‐infused BC films showed mildew and stem shedding with a moldy rate of 100% for 10 tested samples (Figure S13a, Supporting Information). Tomatoes packaged with essential oil‐infused BC films remained free of mold. The weight losses for tomatoes packaged in palm oil and the ones packaged in essential oil were both maintained below 3% after 7 days of storage, implying their WVTRs were similarly low (Figure S13b, Supporting Information). These results suggest that both contents contributed to the low permeability of OBC, while the essential oil that contained thymol molecules was responsible for inhibiting microbial together with cinnamaldehyde molecules.

The antimicrobial function of OBC and PE was compared by observing the growth of fungi microorganisms (Figure 2f). The exemplary fungi species‒*Botrytis cinerea* grew to a large area around the PE film, but failed to reproduce around the OBC film. The radius of the inhibition circle reached 42 mm for the OBC film with a radius of 10 mm, confirming the high antifungal performance of OBC.

To thoroughly reveal the biosafety information of the OBC film, we performed a series of cytotoxic experiments by cell analysis and residue detections by gas chromatography (GC). For L929 mouse fibroblast cells, pristine BC film, palm oil, and a mixture of thymol and palm oil with a volume ratio of 1:1 were not cytotoxic (Figures S14–S17 and S20, Supporting Information). Adding 10 vol% of cinnamaldehyde made palm oil and the OBC film cytotoxic (Figures S18–S20, Supporting Information). However, in the OBC package, cinnamaldehyde was gradually evaporated out and functioning in the gas phase, meaning cinnamaldehyde residue was unlikely to be left on the surface of strawberries. As shown in Figure 2g, no traces of cinnamaldehyde and thymol were detected by GC on the surface of strawberries after 72 h of storage in an OBC‐sealed container, suggesting that the packaged fruits were free from the odor of cinnamaldehyde. For a strawberry in contact with OBC, no GC peaks of cinnamaldehyde and thymol were identified after washing strawberries with water (Figure 2g; Figures S21 and S22, Supporting Information). The cinnamaldehyde and thymol signals were clearly captured on the standard sample composed of 10 mL acetone and 0.1 µL oil mixture (palm oil: essential oil: cinnamaldehyde = 5:5:1), corresponding to ≈0.01 mg of cinnamaldehyde. The comparison between GC signals for the strawberry and standard sample gave rise to the conclusion that each strawberry exposed to OBC contained far < 0.01 mg of cinnamaldehyde. The approved dosage of cinnamaldehyde in food or pharmaceuticals was 1.5 mg per 1 kg body weight by the European Union and U.S. Food and Drug Administration.^[^ ^48^, ^49^, ^50^ ^]^ These results suggest the OBC film is safe to use in food packages. Furthermore, the oil composition is broadly tunable from no cinnamaldehyde to cinnamaldehyde‐rich oils depending on whether the application scenario necessitates strong antimicrobial function or not.

Depending on the antimicrobial activity requirement of the food being packaged, different antimicrobial agents were added to OBC. Meats and strawberries are prone to mold,^[^ ^51^, ^52^ ^]^ so both cinnamaldehyde and thyme essential oils were included in the OBC film to access a high antimicrobial activity. Since tomatoes prefer a moderate antimicrobial activity,^[^ ^43^ ^]^ the OBC film for tomato storage contained only thyme essential oils.

**Figure 3:**
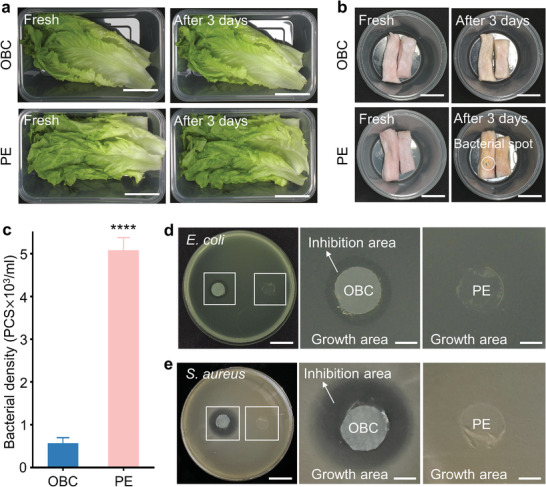
Application of OBC in vegetable and meat packaging. a,b) Photographs of lettuces (a) and a piece of pork (b) before and after being stored in OBC‐ and PE‐sealed containers at 23 °C for 3 days. Scale bars are 4 cm in (a) and 3 cm in (b). c) Density of the *Escherichia coli* (*E. coli*) bacteria in shrimps packaged by OBC and PE at 23 °C for 1 day. The data is a qualitative reflection obtained by a bacterial value‐added assay, not the absolute density of *E. coli*. ^∗∗∗∗^ *p* < 0.0001 by Two‐way ANOVA, compared with the response of the OBC group. d,e) Comparison of the antibacterial property of OBC and PE using *E. coli* (d) and *Staphylococcus aureus* (*S. aureus*) (e), which are two common bacteria that cause the spoilage and poisoning of meat, respectively. Scale bars are 2 cm for the left column and 5 mm for the middle and right columns.

Leafy vegetables (e.g., lettuce and spinach) are examples that can get rid of cinnamaldehyde since they are less susceptible to mold compared to fruits when stored at room temperature. Nevertheless, these vegetables need to inhibit the evaporation of water from the leaves, resulting in a high demand for the barrier property of the package materials. The OBC film with pure palm oil delivered comparable performance with PE when storing lettuce and spinach at room temperature for 3 days, both achieving significantly higher freshness compared with the open condition (**Figure** **3**a; Figure S23, Supporting Information). Weight retention of OBC‐ and PE‐packaged lettuce were 85.2% and 86.5% after 3 days at 23 °C, respectively, confirming the similar preservation performance of PE and OBC for lettuce (Figure S24, Supporting Information).

Fresh meat requires both antibacterial and barrier functions to prevent spoilage and oxidation, respectively. A piece of pork packaged by PE at room temperature for 3 days generated a black bacterial spot and underwent oxidation‐induced yellowing, while the pork packaged by OBC had no black plaque and showed a reduced yellowing effect (Figure 3b). Similar inhibitions of bacterial spots and oxidation were observed on OBC‐packaged shrimps (Figure S25, Supporting Information). The oxidation inhibition originated from the low OTR of OBC, which prevented outside oxygen from entering the package (Figure 1f). The inhibition of bacteria's influence was a direct result of the antibacterial function of the active components (i.e., cinnamaldehyde and thymol) released from OBC. The density of *Escherichia coli* (*E. coli*) bacteria in OBC‐packaged shrimps (567 mL^−1^) was order‐of‐magnitude lower than that of PE‐packaged shrimps (5076 mL^−1^, Figure 3c). The propagation of common meat pathogens, such as *E. coli* and *Staphylococcus aureus* (*S. aureus*), was indeed inhibited by the OBC film but not the PE film, confirming the antibacterial function of the OBC film (Figure 3d,e). In general, the characteristics of low WVTR and OTR, antifungal, antibacterial, transparent, and stretchable made OBC a versatile packaging material that delivers otherwise‐inaccessible storage performance for fresh foods, including fruits, vegetables, and meats.

**Figure 4:**
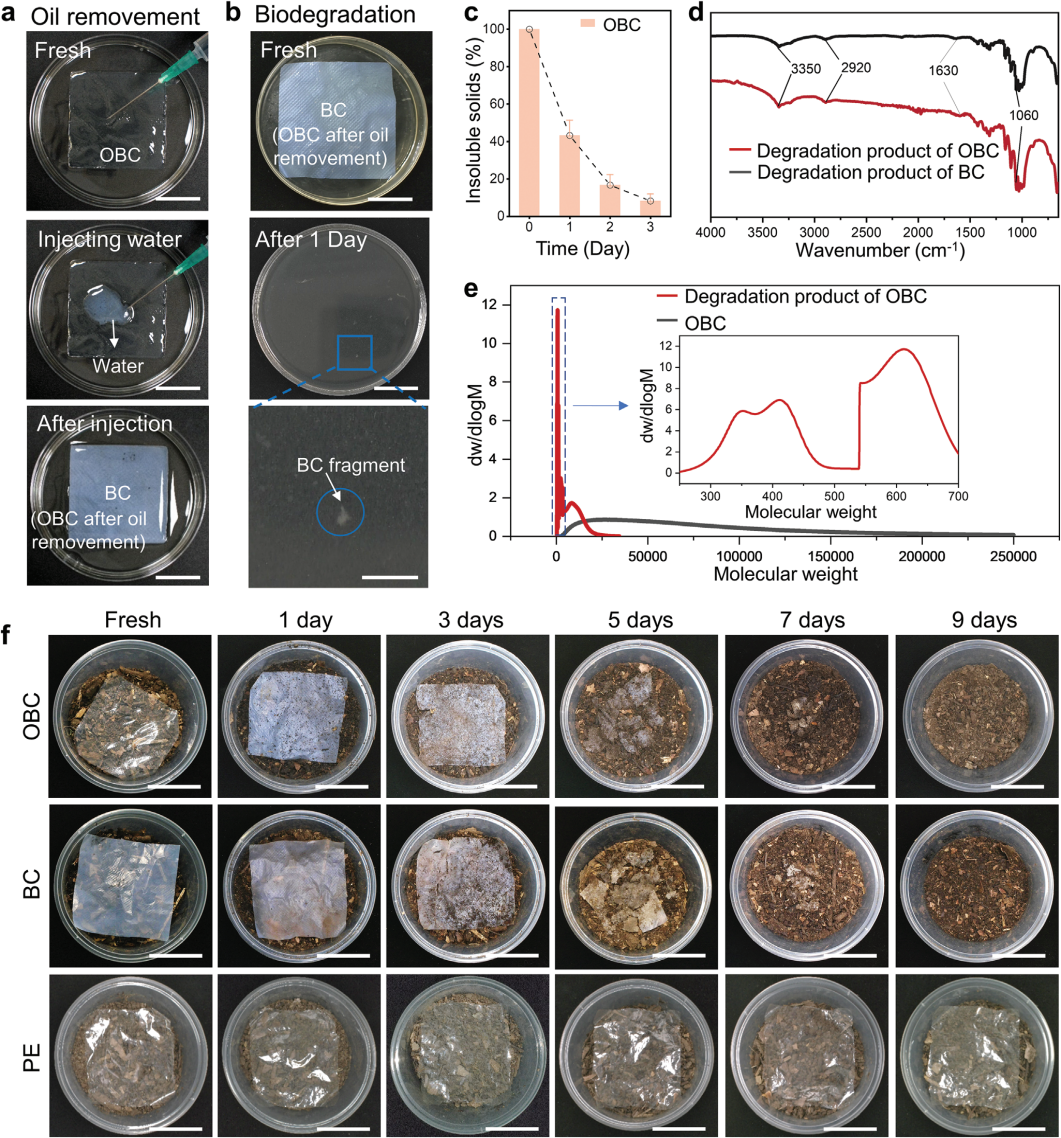
Biodegradation properties of OBC in cellulase solution and natural moist soils. a) Photographs showing the removal of oil from OBC by water. The film turned from transparent to white as a result of the out‐diffusion of oil that made the OBC back to porous BC. Scale bars are 4 cm. b) Photographs showing the biodegradation of OBC in an aqueous cellulase solution at 23 °C. Scale bars for the top two and the bottom pictures are 4 cm and 1 cm, respectively. c) Mass variation of the insoluble solids as a function of the degradation time for an OBC film placed in an aqueous cellulase solution at 23 °C. d) FTIR spectra for the degradation products of OBC and BC films. e) Distributions of molecular weight for OBC films before and after biodegradation in an aqueous cellulase solution. f) Photographs comparing the degradation of OBC, BC, and PE films in natural moist soils at 30 °C. Scale bars are 4 cm.

The biodegradability of OBC is comparable with that of BC. The oil components used in the OBC design (i.e., palm oil, thyme essential oil, and cinnamaldehyde) are all naturally degradable liquids with molecular weights < 500, meaning the BC component (with a molecular weight of 10 000 to 1 000 000) is the limiting step that determines the degradation rate. The dynamic feature of liquid allowed the infused oil to be removable by water, exposing the BC fiber surface to the environment. As shown in **Figure** **4**a, placing a water droplet on the OBC surface triggered an out‐diffusion of the oily liquid and turned the film back to the porous status like a pristine BC film. The abruptly increased opacity was clear evidence of the film's structural change from oil‐infused to porous states. The OBC film with oil being partially removed was degraded into small BC fragments after immersing in an aqueous cellulase solution at 23 °C for 1 day (Figure 4b). The content of the undegraded BC fragments gradually decreased from 43.2% at day 1 to 8.2% at day 3 (Figure 4c).

The degradation product of OBC was identical to that of BC: both were saccharides, as characterized by the absorption peaks on Fourier‐transform infrared spectroscopy at 3350, 2920, 1630, and 1060 cm^−1^, corresponding to the stretching vibrations of O─H, C─H, C═O, and C─O, respectively (Figure 4d). The molecular weight distribution of OBC was changed from 2500–250,000 to 300–700 after 4 days of biodegradation in the enzyme solution, further verifying the fast degradation of OBC (Figure 4e). The infused oils and degradation product ‒ polysaccharides can be recycled by solution methods, potentially allowing the OBC to be repeatably used.^[^ ^53^, ^54^ ^]^

The natural degradation rate of OBC was evaluated by burying the film in moist soil at 30 °C. As shown in Figure 4f, the OBC film was fully degraded on day 9, similar to the degradation rate of BC under the same condition. In contrast, the PE film had no signs of degradation. The mass variation of OBC, BC, and PE films as a function of the degradation time further highlights the fast degradation speed of OBC and BC (Figure S26, Supporting Information). Adding cellulase solution to the soil could reduce the degradation period to 4 days (Figure S27, Supporting Information). In contrast, hydrophobic degradable food packaging materials (e.g., poly(lactic acid)) typically require more than 60 days of industrial composting at 50 °C.^[^ ^55^ ^]^ Petroleum‐based food packaging plastics (e.g., PE) commonly require hundreds and even thousands of years to completely degrade.^[^ ^56^ ^]^ The mild condition and fast speed make OBC a highly environmentally friendly material for food packaging.

The liquid seal strategy applies to a variety of porous cellulose films, such as wood pulp papers and butter papers. The freshness of strawberries stored in oil‐infused pulp and butter papers was apparently higher than that of strawberries stored in pristine papers (Figure S28, Supporting Information). Although these papers have different intrinsic degradation rates in the moist soil (20 and 15 days for wood pulp and butter papers, respectively), the oil infusion did not change their degradation behaviors (Figures S29 and S30, Supporting Information). Wood pulp and butter papers are cheaper than bacterial cellulose, providing a low‐cost option for oil‐infused degradable packaging design.

## 3 Conclusion

In summary, we developed a fully biodegradable packaging material with high food‐keeping performance based on an oil‐infused bacterial cellulose (OBC) film. The newly introduced oil infusion strategy favorably altered the optical, mechanical, transport, and antimicrobial properties of cellulose films towards an effective packaging material, without decreasing cellulose's intrinsically high degradation rate. The optical transparency at a wavelength of 500 nm and the elongation at break were both improved by 3.4 times for the cellulose film after oil infusion, making the cellulose easier to use during food storage. The WVTR and OTR were decreased by ≈50% and 3 orders of magnitude, respectively, resulting in a substantial reduction of water loss for fruits and vegetables as well as an effective inhibition of meat oxidation. Based on the antifungal molecules that were released from the oil content, the strawberries stored by OBC showed a moldy rate of 0%, a remained nutrient content of 73%, a remained weight of 96.9%, and a remained firmness of 54.6% after 5 days of storage at 23 °C, significantly outperforming the strawberries stored by BC and PE. As a result of the low OTR and antibacterial properties, OBC‐stored pork showed no visible oxidative yellowing and black spots after 3 days at 23 °C. The OBC‐stored shrimp exhibited a bacterial density of 567 bacteria mL^−1^ after 12 h at 23 °C, corresponding to a 90% reduction compared with the one packaged by commercial PE. The OBC‐stored leafy vegetables were as fresh as those stored by PE. The oil in the OBC film diffused out of the film in the water environment, allowing the OBC film to switch back to a BC film after use. As a consequence, the OBC film was completely biodegraded after 9 days of burying in naturally moist soil at 30 °C, which was comparable to the degradation rate of BC. With its general applicability to other porous films, the oil infusion strategy provides broad implications for designing biodegradable packaging materials.

## 4 Experimental Section

### Materials

Palm oil, cinnamaldehyde, and anhydrous copper sulfate were purchased from Aladdin's Reagent Co., Ltd. Thyme essential oil (thymol content > 40%) was purchased from Shanghai Yuanye Biotechnology Co., Ltd. Bacterial cellulose (BC) hydrogel films were purchased from Guilin Qihong Technology Co. Ltd. UV curing adhesive (NOA63) was purchased from Norland Inc. Cellulase solution (derived from *Trichodermareesei*, 25 KU) and acetone (chromatographically pure) were purchased from Sigma‐Aldrich Inc. Strawberries and cherry tomatoes were purchased from fruit stores in Shenzhen. Lettuce, spinach, pork, shrimp, and poly(ethylene terephthalate) (PET) plastic boxes were purchased from supermarkets in Shenzhen. Poly(ethylene) (PE) films were purchased from Canon Inc. *Botrytis cinerea*, *Escherichia coli*, *Staphylococcus aureus*, and L929 mouse fibroblast cells (L929 cells) were purchased from BeNa Culture Collection Co., Ltd. Phosphate buffer solution (PBS), minimum essential medium (MEM), fetal bovine serum (FBS), penicillin‐streptomycin solution (P/S), and trypsin solution were purchased from Thermo Fisher Scientific Inc. Live/Dead cell staining kit, CCK‐8 kit was purchased from Shanghai Biyuntian Biotechnology Co., Ltd. Wood pulp papers, butter papers were purchased from a local store in Shenzhen. The soil was excavated from the campus of Southern University of Science and Technology in Shenzhen.

### Fabrication of Oil‐Infused Bacterial Cellulose (OBC) Films

The OBC was prepared according to a previous liquid‐solid composite method.^[^ ^57^ ^]^ The oil was a mixture of 20 mL palm oil, 20 mL thyme essential oil, and 4 mL cinnamaldehyde (i.e., the volume ratio of palm oil: thyme essential oil: cinnamaldehyde was 5:5:1). Prior to the infusion, the oil mixture was stirred in a conical flask for 2 h at a speed of 500 r min^−1^. Bacterial cellulose hydrogel films with a thickness of 0.3 mm were placed in a 50 °C oven for 12 h to obtain the dry bacterial cellulose films with a thickness of 15 µm. The dry bacterial cellulose films were soaked in the oil mixture at room temperature for 2 h to obtain the OBC film. The excess oil on the surface of the OBC film was wiped off with dust‐free paper before usage. The oil content was determined by weighing the mass of BC film before and after the oil infusion.

### AFM

Atomic force microscopy (AFM) images were obtained using an Asylum MFP‐3D system under tapping mode. AC240TS cantilevers (Olympus) were used to record AFM images (sizes ranging from ≈1 to ≈400 µm^2^) at an imaging force of ≈2 N with a resonance frequency of 70 kHz. The sample was scanned with 256 × 256 pixels at a frequency of 0.25 Hz.

### SEM

Scanning electron microscopy (SEM) images were acquired on Hitachi SU8230 field‐emission scanning electron microscopes. The accelerating voltage and electron beam current were 3 kV and 10 nA, respectively. To avoid charging effects, a thin Pt film was deposited on the surface by ion sputtering before SEM observation.

### XRD

X‐ray diffraction (XRD) was performed on the Rigaku Smartlab X‐ray diffractometer. The X‐ray was a monochromatic Cu Kα ray with a power of 200 W (10 mA, 20 KV).

### Confocal Laser Scanning Microscope

Confocal fluorescence images were obtained on LSM 980, Zeiss. Prior to the measurement, BC films were stained by an aqueous solution of juanoyl rhodamine B (red under fluorescence microscopy with an excitation wavelength of 543 nm). The stained BC films were placed in a 50 °C oven for 12 h to remove moisture. Then, the dry BC films were immersed in an oily liquid containing coumarin 6 (green under fluorescence microscopy with an excitation wavelength of 488 nm) for 2 h. The final OBC films were obtained with BC fibers stained with juanoyl rhodamine B and oily liquid stained with coumarin 6. Since the microscope is inverted (objective lens below the sample), the sample was held in place by a coverslip. At 20X objective magnification, the fluorescence microscope was controlled by the Z‐stack mode of the Zeiss ZEN software to take multiple sets of fluorescence micrographs along the z‐axis direction with a scanning step of 1.2 µm from top to bottom. X‐axis and y‐axis positions remained unchanged. Each image was taken with a pixel resolution of 1024 × 1024. 3D images were reconstructed using ZEN software.

### Water Vapor Transmission Rate (WVTR)

The WVTR of BC, OBC, and PE films were measured using the weighing method based on previous descriptions and improvements to the experimental methodology.^[^ ^58^ ^]^ In specific, 7 mL of anhydrous copper sulfate powder as the hygroscopic agent was placed in a 10 mL vial. The mass of the vial containing anhydrous copper sulfate was weighed and noted as W_1_. The vial mouth was subsequently sealed by the testing film using a UV‐curable adhesive as the glue. The vial capped with the testing film was placed at 25 °C and 70% relative humidity for 2 days. After that, removing the UV‐cured adhesive testing film from the vial, and weighing the mass of the vial containing hydrated copper sulfate, noted as W_2_. If denoting the inner diameter of the vail mouth as D, the WVTR was calculated according to the following equation:

(1) $$WVTR=\left(W_{2}-W_{1}\right)/\left(2\times 0.25\times \pi \times D^{2}\right)$$

### Oxygen Transmission Rate (OTR)

OTR was measured by an oxygen permeation analyzer (OX‐TRAN 2/22H, AMETEK MOCON). The measurement was conducted at 25 °C with 70% relative humidity and 0.01 MPa oxygen partial pressure. For the OBC film, pure palm oil was used to avoid the contamination of equipment caused by volatile substances (i.e., cinnamaldehyde and thymol).

### Optical Transparency

Optical transparency was measured on an ultraviolet and near‐infrared spectrophotometer (Lambda 950). During the test, the film was directly placed on the test rig, and the light wavelength was set from 250 to 800 nm.

### Mechanical Property

Stress‐strain curves of BC, OBC, and PE films were acquired on an electromechanical universal testing machine (CMT6203, MTS SYSTEMS Co., Ltd). The films had a film thickness of 15 µm and were cut into dumbbell‐shaped specimens with a width of 4 mm before the measurement. The stretching speed was set to 20 mm min^−1^.

### Wetting Property

The wetting property of BC film was evaluated by water and oil contact angle measurements. Water and oil contact angles on the film were recorded by a contact angle goniometer (KRÜSS DSA25) at ambient conditions and analyzed by ADVANCE software. The droplet volume was 100 µL for both water and palm oil.

### Sample Preparation for Fruit Storage Evaluation

In reference to previous work,^[^ ^43^ ^]^ fruits with comparable freshness were selected for the evaluation. Strawberries and cherry tomatoes used in the experiment had a mass of 30–40 g and 8–12 g per unit, respectively. All fruits were washed in deionized water for 5 min to remove impurities and then blotted with clean paper towels to remove water. The dry fruits were placed in commercial PET boxes with a capacity of 500 mL. Cut a 4.5 cm × 4.5 cm square opening hole in the lid of the PET box. The tested BC, OBC, or PE films with a size of 5 cm × 5 cm were used to cover the hole. The film edge and PET lid were glued together by a UV‐curable adhesive. The fruits packaged inside the PET box capped by the specially designed lid were placed at 23 or 3 °C for the storage test. Different samples were named according to the testing films on the lid hole. For example, an open container refers to no testing film on the hole, OBC‐sealed container refers to OBC covering the hole.

### Moldy Rate Count for Fruits

The moldy rate was counted as described in a previous study.^[^ ^43^ ^]^10 fruits, evenly placed in 5 containers with the same testing film, were used to count the moldy rate (i.e., 2 fruits in 1 container). The number of moldy fruits was recorded every day. The ratio between the moldy fruits and the total fruits was the moldy rate. The moldy rate presented in the manuscript was an average result of three groups of experiments (30 fruits in total).

### Total Soluble Solids (TSS) Content for Fruits

TSS was measured by extracting a few drops of juice from the fruit and analyzing the drops with a handheld refractometer (RAB‐18, AS ONE) at 20 °C.

### Weight Loss Measurement

The initial weight of the fruit was recorded as W_0_. The weight of the fruit after n days of storage was recorded as *W*_n_. Weight loss was calculated by the following equation:

(2) $$\mathrm{Weight}\;\mathrm{loss}=\left(W_{0}-W_{n}\right)/W_{0}\times 100\%$$

### Firmness Measurement

A fruit firmness tester (SN‐FHD006, Sunne) was employed for the measurements.^[^ ^44^ ^]^ The tester used a pressure head with a diameter of 3 mm to press the strawberry until the skin of the strawberry breaks. The maximum value of the force exerted by the pressure head was recorded as the firmness of the strawberry at the region being pressed. Each strawberry was randomly pressed in three different regions, and the average firmness of the three regions was taken as the firmness of the strawberry. The firmness of 10 strawberries was measured to improve the measuring accuracy. For the cherry tomato case, the firmness was obtained by a universal mechanical testing machine (CMT6203, MTS SYSTEMS Co., Ltd), following a standard method.^[^ ^59^ ^]^ The cherry tomatoes were pressed by the testing machine at a speed of 5 mm min^−1^ until they ruptured. The maximum pressure exerted by the machine was recorded as the firmness of the cherry tomato.

### The Storage Performance Test for Meats and Vegetables

Following a similar process to the fruit case.

### Detection of Residual Antimicrobial Components on the Surface of Strawberries

Gas chromatography (GC) was employed to deliver this task.^[^ ^60^ ^]^ A strawberry was washed repeatedly with 10 mL of acetone for 5 min to fully extract the soluble substances on the surface. The acetone was collected and analyzed by an Agilent 7890B‐5977A gas chromatography‐mass spectrometry (GC‐MS) instrument with an injection volume of 1 µL, a split ratio of 1:5, a column temperature range of 60 to 290 °C, and a temperature increase rate of 10 °C min^−1^. For strawberries that were directly contacted with OBC (Figure S21, Supporting Information), the GC measurement was performed after 1 day of contact. In Figure 2g, the sample “Physical contact with OBC after washing” was cleaned with deionized water for 2 min before GC measurement. The OBC oil/acetone standard solution was obtained by dissolving 0.1 µL of the oil infused in OBC into 10 mL of acetone.

### Measurement of the Oxygen Content in the Sealed Container

The oxygen content in the OBC‐caped PET container with strawberries inside was measured by an oxygen content testing tube (31B, Asone). A syringe was used to extract the air inside the PET container and inject the air into the oxygen testing tube, whose other end connected to the PET container. The air was circulated between the container and the testing tube over 30 times to improve the accuracy of the oxygen testing tube. The measurement was repeated three times and the average value recorded by the oxygen testing tube was used.

### Measurement of Volatile Gas Composition of OBC Films

Volatile gas composition was performed on a Thermo ISQ 7000 GC‐MS equipment loaded with a headspace injection unit. Similar to the fruit storage experiment, the OBC film with a size of 5 cm × 5 cm was placed on a square opening (4.5 cm × 4.5 cm) on the lid of the PET container. After one day of natural evaporation at 23 °C, the gas in the PET container was extracted for GC measurement.

### Measurement of Residual Content of Antimicrobial Agents in OBC Films

Thymol content was selected as the representative for determining the remaining amount of the antimicrobial active substance in OBC. The OBC films with a size of 4 cm × 4 cm were left exposed at 23 or 3 °C for volatilization of the active substance. After being left for the specified number of days, the films were immersed in 10 mL of N‐pentane for 3 days to dissolve the thymol molecules. The absorbance of the N‐pentane solution was measured at 275 nm using a UV spectrophotometer. The absorbance at 275 nm reflects the thymol content in the OBC film.^[^ ^44^ ^]^

### Antifungal Test

Antifungal experiments were performed on an ultra‐clean bench based on standard experimental methods.^[^ ^61^ ^]^ The fungus used in the experiment was a common fungus that causes mold in strawberries: *Botrytis cinerea*. The culture medium was composed of potato cooking liquid 1 L, glucose 20 g, KH_2_PO_4_ 3 g, MgSO_4_·7H_2_O 1.5 g, vitamin B1 10 mg, agar 20 g, pH = 6.0 ± 0.2. Potato cooking liquid was prepared by boiling 300 g of peeled potato pieces in water for 30 min and collecting 1 L of filtrate. The culture medium was sterilized at 121 °C for 15 min before use. The cooled medium was reheated to 90 °C to be liquefied and then poured into a circular petri dish until the medium spread over the bottom of the dish. After the medium was cooled to room temperature, the fungal solution containing *Botrytis cinerea* was evenly coated on the surface of the medium to inoculate the fungus. To prevent direct contact between testing films and fungus, two coverslips with a diameter of 1 cm were placed on the medium for positioning OBC film and PE film of the same size (Figure 2f). The petri dish with a lid was placed in an incubator at 28 °C for 7–14 days to culture the fungus.

### Bacterial Value‐Added Assay for Shrimp

In a sterile environment, 5–10 mg of shrimp meat stored by OBC or PE film was placed in a liquid bacterial medium and incubated at 37 °C. The liquid bacterial medium was composed of 1 g peptone, 0.5 g yeast powder, 1 g NaCl, and 100 mL deionized water. The medium was sterilized at 121 °C for 15 min before use. After 12 h of incubation, 100 µL of the culture solution was transferred to a 96‐well plate and the optical density values (noted as D) of the culture solution at 600 nm were tested by a microplate reader (BioTek Instruments, Inc.). Calculate the density of *E. coli* according to the following equation:

(3) $$\mathrm{Density}\;\mathrm{of}\;E.\;coli=0.87087e^{36.43D}$$

### Antibacterial Test

The solid bacterial medium was obtained by adding 2 g of agar into 100 mL of the liquid bacterial medium used in the bacterial value‐added assay for shrimp. The bacteria used in this test were *E. coli* and *S. aureus* (used independently). The procedure was similar to that for the antifungal test, except the incubation condition was 37 °C for 24 h.

### Culture of L929 Cells

All experimental operations were performed in a sterile environment (clean room, ultra‐clean bench). The cell culture medium (liquid) was composed of MEM Alpha medium, fetal bovine serum (PBS), and penicillin‐streptomycin solution (P/S) with a volume ratio of 9:1:0.1. The frozen cells were placed in a water bath at 37 °C for 1 min, together with the frozen tubes for resuscitation. 1 mL of thawed cell suspension was transferred into a sterile centrifuge tube with a pipette gun. 2 mL of cell culture medium was added to dilute the dimethyl sulfoxide (DMSO) in the freezing solution (DMSO is harmful to cells). To completely remove DMSO, the tube containing 1 mL of cell suspension and 2 mL of cell culture medium was centrifuged at 1200 r min^−1^ for 3 min. All liquid in the centrifuge tube was poured out after centrifugation, leaving the cell mass at the bottom of the tube. 1 mL of cell culture medium was added to the tube, and the cell agglomerates were blown away by a pipette gun. After that, a new cell suspension was obtained with the cells evenly distributed in the culture medium. 300 µL of the new cell suspension was transferred into a round petri dish with a diameter of 9 cm, together with 9 mL of cell culture medium. The dish was gently shaken to homogenize the cells. Covering with a lid, the dish was placed in a cell culture incubator for cultivation (culturing condition: 37 °C, 5% of CO_2_ + 95% of air). The culture medium was changed every other day.

After the cells occupied 80% of the dish surface, the cell passaging operation was performed. Since L929 cells were wall‐adherent cells, the passaging process needed to release the wall‐adherent state of cells. The cell culture solution in the petri dish was directly poured off, then used 2 mL of PBS solution to wet wash L929 cells once to remove the residual cell culture medium. A total of 1 mL trypsin solution (trypsin content of 0.25%) was further added to the petri dish and placed the dish with a lid into the cell culture incubator for 1 min at 37 °C. After the incubation, rounding of the cells was observed under a light microscope, indicating that the cells had been released from the adherent state. At this point, the cells were still adhering to the wall of the petri dish, but the adhesion was weakened. A total of 2 mL of cell culture medium was further added to the petri dish and washed off the cells from the walls of the petri dish with a pipette gun. All the liquid in the dish was subsequently collected into a centrifuge tube and centrifuged at 1200 r min for 3 min. After removing the liquid, the cell clumps were left at the bottom of the centrifuge tube and trypsin (detrimental to the cells) was eliminated. The following procedures were identical to those performed after centrifugation in the cell resuscitation. The cell culture – cell passaging – cell culture operation was repeated until the number of passages reached 9, which was the condition that the cells began to lose their ability to proliferate.

### Cell Live‐Dead staining Assay

Cell live‐dead staining assays were performed on bacterial cellulose film, palm oil, palm oil + thyme essential oil (1:1, v/v), palm oil + cinnamaldehyde (10:1, v/v), and palm oil + thyme + cinnamaldehyde (5:5:1, v/v). All operations were performed in a sterile environment, except for the final fluorescent micrographs. The well plate used for the experiment was a 48‐well plate, and the diameter of each well in the plate was 10 mm. All experimental materials were sterilized before the experiment. The cellulose film was sterilized by autoclave (121 °C, 0.11 MPa) and the oily liquid was sterilized by screening out the microorganisms using a filter with a diameter of 200 nm. For the film sample, a film was directly placed with a diameter of 10 mm at the bottom of the holes of the plate. For the oily liquid (i.e., thyme essential oil, cinnamaldehyde, and palm oil), a total of 1 µL of the sterile oily liquid was used to uniformly cover the bottom of the wells.

During the experiment, three well plates were employed with six wells used on each. Three wells of each well plate were placed with the same sterilized materials as the experimental group and another three wells were selected without any material as the blank control group. After placing the material, a cell suspension (containing 10,000 L929 cells per 700 µL of cell suspension) was transferred into the control wells and experimental wells. The cells were implanted above the experimental material. After that, the well plates were placed in an incubator for cell culturing (culture conditions: 37 °C; 5% CO_2_ + 95% air). The three well plates were cultured for 1, 3, and 5 days, respectively. The culture medium was changed every other day during the incubation.

After the incubation, a live‐dead staining operation was performed and followed by fluorescence micrographs (Figures S14–S19, Supporting Information). The dyes used for live‐dead staining were Calcein AM and Propidium Iodide. Calcein AM (fluorescence excitation at 405 nm) stained live cells with green fluorescence and Propidium Iodide (fluorescence excitation at 543 nm) stained dead cells with red fluorescence. The staining solution was configured to the volume ratio of Calcein AM: Propidium Iodide: PBS solution of 1:1:1000. Before staining, the cells were rinsed with PBS solution. After that, 300 µL of staining solution was added to each well. The well plate was lastly placed in the cell incubator for 30 min. Then, the fluorescence micrograph of the cells was taken by a confocal fluorescence microscope.

### Relative Cell Numbers Measurement

Relative cell number measurements were conducted by the CCK‐8 assay. Before staining the cells with Calcein AM and Propidium Iodide, the experimental procedure of the CCK‐8 assay was the same as that of the cell live‐dead staining assay. The CCK‐8/cell culture medium solution with a volume ratio of 1:10 was added to the experimental and control wells with an amount of 300 µL per well. The wells were placed in an incubator for 2.5 h to induce the cells to produce sufficient formazan. Formazan is a cellular product that can indicate the number of cells by controlling the light absorbance of cell cultures at 450 nm. After the incubation, the cell culture medium in the wells was transferred one by one to a 96‐well plate, and their optical density (OD) values at 450 nm were measured by a microplate reader. Cell viability was obtained according to the following equation:

(4) $$\mathrm{Relative}\;\mathrm{cell}\;\mathrm{numbers}=OD_{E}/OD_{0}$$

*OD*_E_: *OD* values are measured after *n* days of incubation. *OD*_0_: *OD* values are measured after one day of incubation for the control groups (blank groups).

### Degradation in an Enzymatic Solution

Degradation experiments were performed regarding previous experiments, and the experimental material was changed from PCL to BC or OBC.^[^ ^62^ ^]^ The OBC film was immersed in a diluted cellulase solution with 5 mL of cellulase solution and 20 mL of water for 1–3 days at 23 °C. The oily liquid in the OBC film diffused out and floated on the surface of the solution due to the amphiphilic nature of BC films. To ensure the accuracy of the experiment, three sets of parallel experiments were conducted at the same time.

### Degradation in Moist Soil

Soil degradation experiments were performed regarding previous experiments.^[^ ^62^ ^]^ The soil came from the campus of the Southern University of Science and Technology. A BC or OBC film with a size of 5 cm × 5 cm was buried in the middle of 400 mL of fresh soil that was loaded into a PET container. 200 mL of water was poured into the soil to moisten it. The container was placed in a 30 °C oven during the degradation experiment. The testing film was photographed every 1–2 days. Before photographing, the soil on top of the film was removed. After photographing, the film was covered with soil again, and the degradation experiment resumed in the 30 °C oven.

### Insoluble Solid Measurement

The content of insoluble BC solid reflected the degradation degree of the OBC film. The mass of the substrate BC that was used to fabricate OBC was weighed and recorded as W_0_. After the enzymatic degradation experiment, all undegraded BC fragments in solution were filtered by a filter paper with a pore size of 0.22 µm. The filter paper and the BC fragments on it were dried in an oven at 50 °C for 12 h. The increased weight of the filter paper was measured to determine the mass of undegraded BC noted as *W*_1_. The insoluble solid was obtained by *W*_1_/*W*_0_ × 100%.

### Chemical Analysis of Degraded Products

The products after degradation were characterized by a Fourier transform infrared (FTIR) spectrometer (Bruker Vertex 70v). Prior to the measurement, the solution containing degradation products of BC or OBC film was centrifuged at 7000 r min^−1^ for 10 min to remove small BC fragments. The oily liquid was removed for the OBC case. The FTIR spectra were acquired by dropping 0.5 mL of the aqueous solution containing the degradation product onto an ATR crystal and setting the resolution of the scan to 4 cm^−1^ and averaging 64 scans. The test background was air.

### Molecular Weight Measurement

Molecular weight measurements were performed on a gel chromatography with a liquid phase system U3000 (Thermo), an oscillometric detector Optilab T‐rEX (Wyatt technology), and a laser light scattering detector DAWN HELEOS II (Wyatt technology), based on a standard method.^[^ ^63^ ^]^ Columns Ohpak SB‐805 HQ (300 × 8 mm), Ohpak SB‐804 HQ (300 × 8 mm), and Ohpak SB‐803 HQ (300 × 8 mm) were used in series in the gel chromatography. For the OBC film (black line in Figure 4e), the sample was dissolved in DMSO (containing 0.5% LiBr, w/w) at a final concentration of 1 mg mL^−1^ and filtered through a 0.45 µm filter for on‐board assay. For the degraded OBC film (red line in Figure 4e), the sample as dissolved in 0.1 mol mL^−1^ aqueous NaNO_3_ solution (containing 0.02% NaN_3_, w/w) at a concentration of 1 mg mL^−1^ and filtered through a 0.45 µm filter for on‐board assay.

### Statistical Analysis

All quantitative data are presented as the mean ± standard deviation. One‐way ANOVA analysis of variance was used to compare three or more conditions, and two‐way ANOVA analysis was used to compare two conditions. Differences with a *p* value of ˂0.05 were considered statistically significant. “^∗∗∗∗^” means *p*<0.0001, “^∗∗∗^” means *p*<0.001, “^∗∗^” means *p*<0.01, “^∗^” means *p*<0.05, “NS” (i.e., not significant) means *p*>0.05.

## Conflict of Interest

The authors declare no conflict of interest.

## Supporting information

Supporting Information

## Data Availability

The data that support the findings of this study are available from the corresponding author upon reasonable request.
