# Supplementary material for: Fully Biodegradable Packaging Films for Fresh Food Storage Based on Oil‐Infused Bacterial Cellulose
Source: Adv Sci (Weinh). 2024 Apr 3;11(23):2400826. doi: 10.1002/advs.202400826 (PMC11187918; doi:10.1002/advs.202400826)
Supplement: Supplementary file 1 — Supporting Information [file ADVS-11-2400826-s001.pdf]

ADVANCED SCIENCE
Open Access

Supporting Information

for *Adv. Sci.*, DOI 10.1002/advs.202400826

Fully Biodegradable Packaging Films for Fresh Food Storage Based on Oil-Infused Bacterial Cellulose

*Guoli Chen, Kaimin Wang, Pinghang Chen, Daohang Cai, Yan Shao, Rui Xia, Chun Li, Haochuan Wang, Fuzeng Ren, Xing Cheng and Yanhao Yu**

Supplementary Information for

Fully Biodegradable Packaging Films for Fresh Food Storage Based on Oil-Infused Bacterial Cellulose

Guoli Chen[1], Kaimin Wang[1], Pinghang Chen[1], Daohang Cai[1], Yan Shao[1], Rui Xia[1], Chun Li[1],

Haochuan Wang[1], Fuzeng Ren[1], Xing Cheng[1], Yanhao Yu[1,2 *]

[1]Department of Materials Science and Engineering, Southern University of Science and Technology, Shenzhen 518055, China

[2]Institute of Innovative Materials, Southern University of Science and Technology, Shenzhen 518055, China

[*] E-mail: yuyh@sustech.edu.cn

Figures

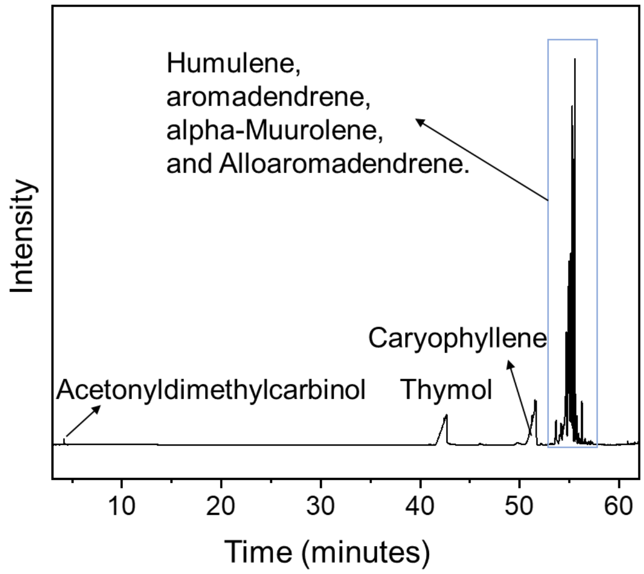

Figure S1. Gas chromatography (GC) of essential oils, showing the essential oils were mainly made of thymol (thymol content > 40 wt%), caryophyllene, humulene, aromadendrene, alpha-muurolene, and alloaromadendrene.

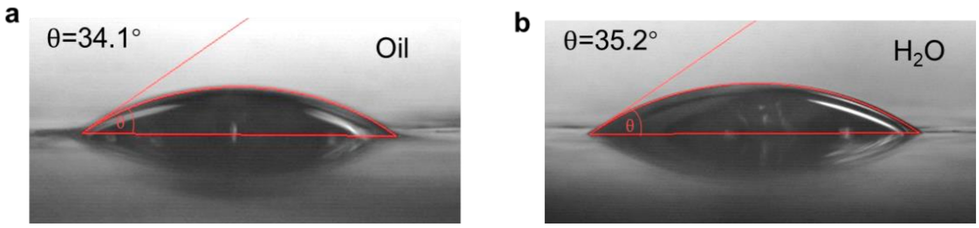

Figure S2. Oil contact angle (a) and water contact angle (b) of bacterial cellulose (BC), indicating that bacterial cellulose was amphiphilic.

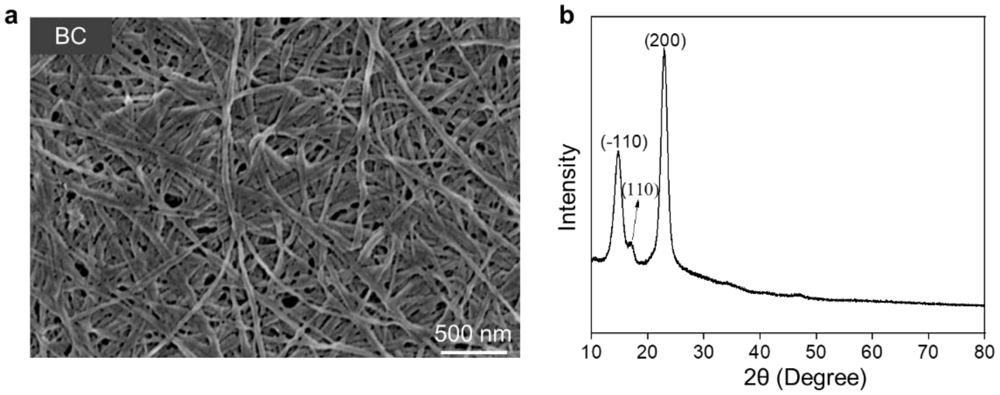

Figure S3. (a) SEM image of BC film, showing a porous structure consisting of entangled fibers with diameters of 10~100 nm and pore sizes in the range of 10~200 nm. (b) XRD characterization of BC film. The peaks at 14.8°, 17.0°, and 22.0° correspond to (-110), (110), and (200) crystal planes of BC crystals, respectively, suggesting the BC film was composed of type I crystals, which were susceptible to biodegradation.

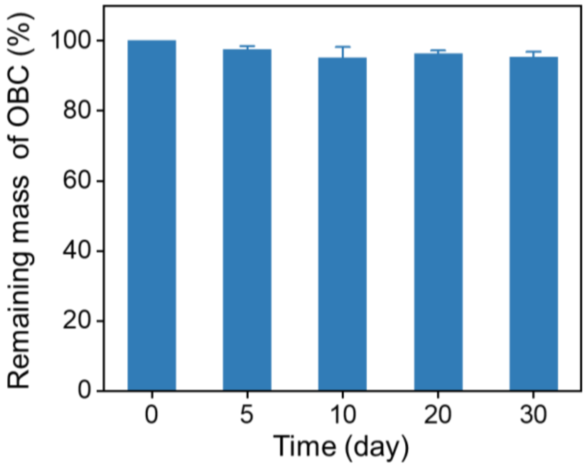

Figure S4. Mass variation of a palm oil-infused BC film as a function of time in an oven at 80 °C.

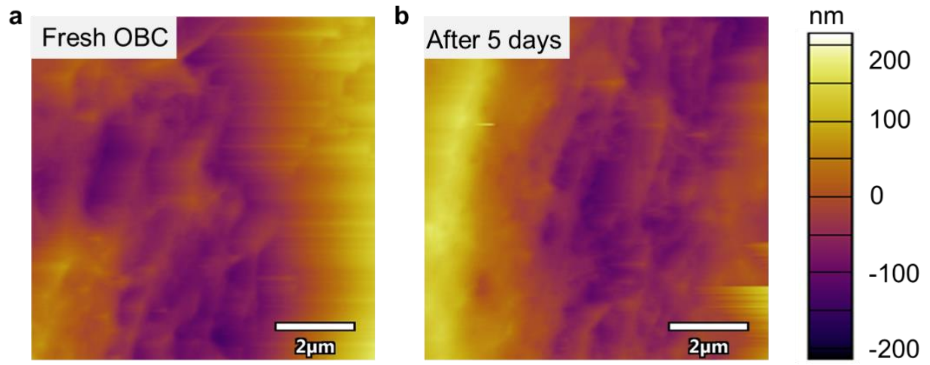

Figure S5. AFM topographic images of the fresh OBC film (a) and after 5 days storage of strawberries (b), showing that the microstructure of the OBC film remained unchanged during food packaging.

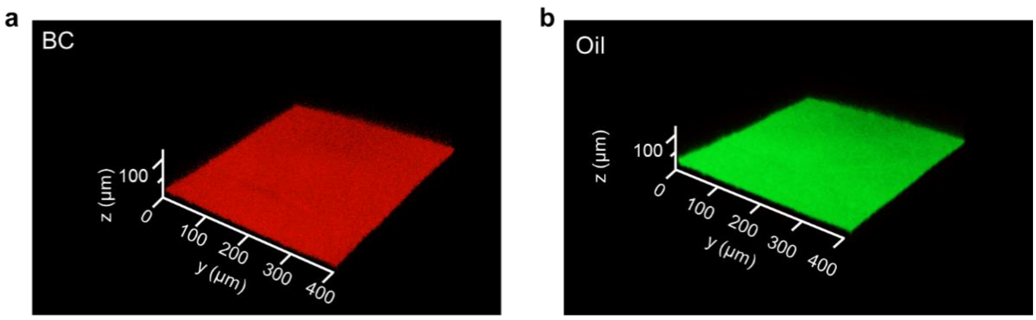

Figure S6. 3D fluorescence microscopy images of BC (a) and oil (b) components in the OBC film, revealing that the oil was uniformly distributed within the BC film.

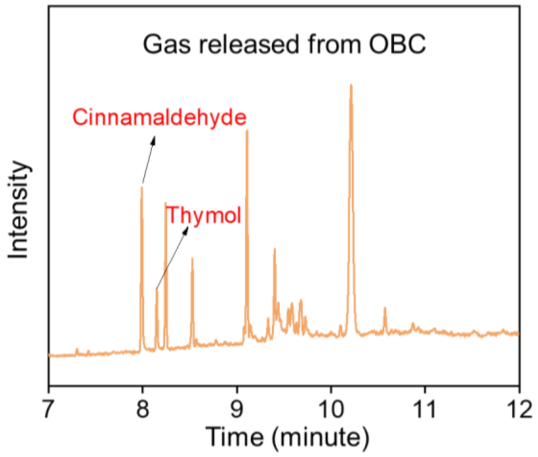

Figure S7. Gas chromatography (GC) of gases in a PET container capped by an OBC film, showing the presence of thymol and cinnamaldehyde (antimicrobial agents) released from OBC. Other unlabeled GC peaks originated from the component of thyme essential oil.

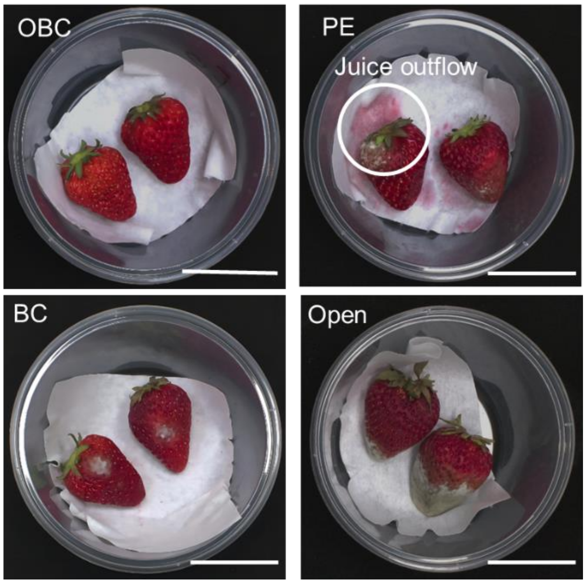

Figure S8. Photographs showing the degree of juice outflow for strawberries stored in an open PET container or a container capped by different films at 23 °C for 5 days. Strawberries capped by a PE film showed the most dramatic juice outflow, presumably caused by the overripening effect. Scale bars are 4 cm.

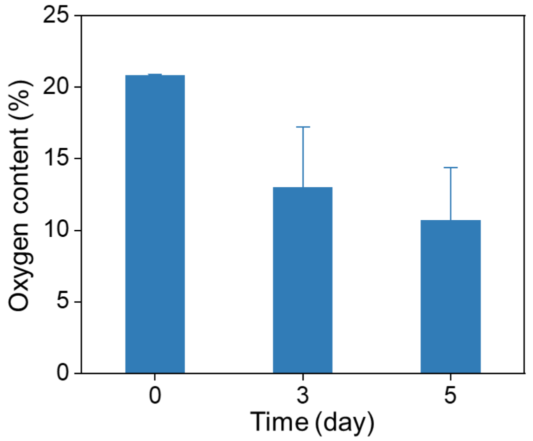

Figure S9. Variation of oxygen content as a function of time in an OBC-sealed container with strawberries stored inside. On the fifth day, the oxygen content was (10%) above the oxygen level (3%) of anaerobic respiration, meaning the strawberries stored by OBC film did not undergo anaerobic respiration. Since the oxygen transmission rate (OTR) of the OBC film is lower than that of PE and BC films (Figure 1f), the oxygen content in the PE and BC cases should be higher than that of OBC. Therefore, no anaerobic respiration occurred in OBC-packaged strawberries indicates no anaerobic respiration occurred in PE-, and BC-packaged strawberries.

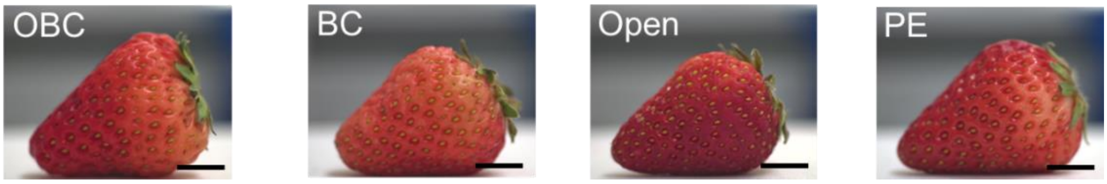

Figure S10. Photographs of strawberries stored in OBC-, BC-, PE-sealed, and open containers after 5 days in a 3 ºC refrigerator, showing no mold generation on all of them at refrigerator condition. Scale bars are 1 cm.

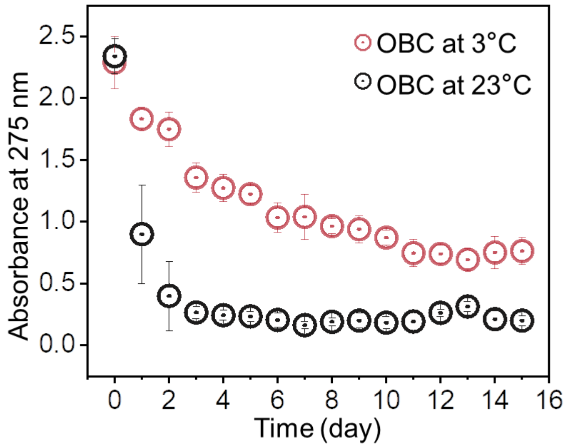

Figure S11. Light absorbance at 275 nm of antimicrobial agent (thymol) in OBC films at 3 ºC and 23 ºC. The absorbance at 275 nm was proportional to the amount of remaining antimicrobial agent in the film. This result indicates that the antimicrobial agent was released slower at refrigerator temperature than that at room temperature, matching the temperature-dependent antimicrobial need for fruit storage.

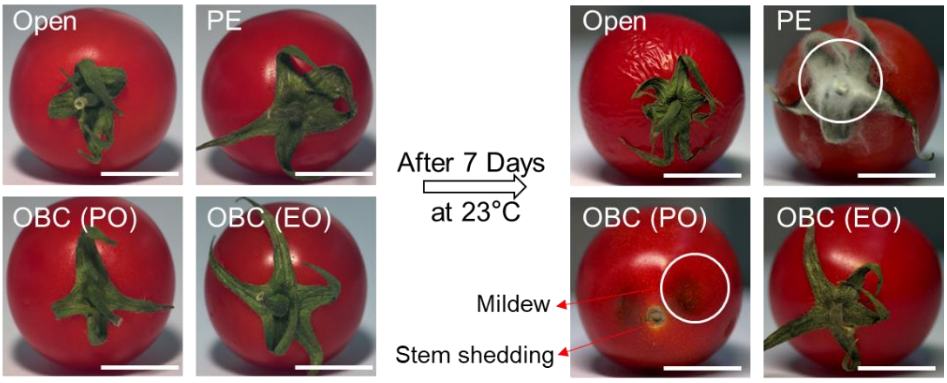

Figure S12. Photographs that compare the appearance changes of cherry tomatoes before and after being stored in open, PE-sealed, OBC (PO)-sealed, and OBC (essential oil (EO))-sealed containers at 23 ºC for 7 days. OBC (PO) refers to palm oil infused bacterial cellulose and OBC (EO) refers to essential oil infused bacterial cellulose. Mold or stem shedding (highlighted in the white circles) was observed in PE-sealed and OBC (PO)-sealed containers due to their low water vapor transmission rates and a lack of antimicrobial function. Cherry tomatoes in Open container exhibited significant water loss. No mold nor water loss was observed in cherry tomatoes stored in the OBC (EO)-sealed container, as a result of the low water vapor transport rate and antimicrobial function of OBC (EO). Scale bars are 1 cm.

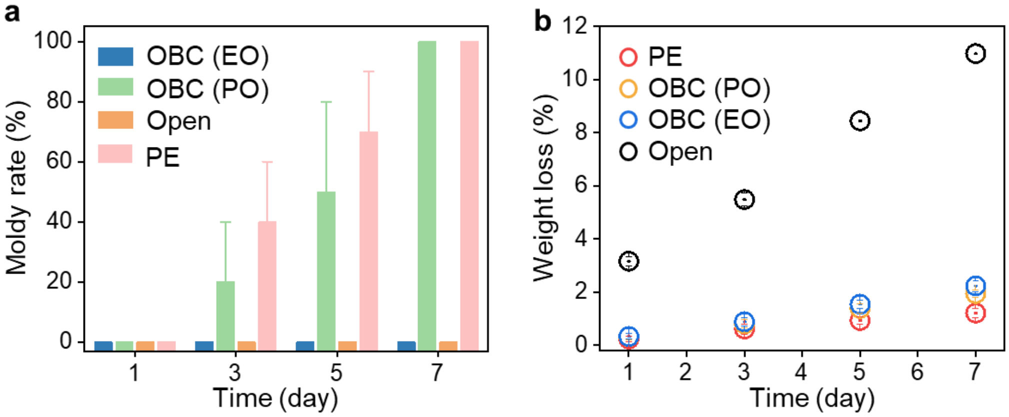

Figure S13. Moldy rate (a) and weight loss (b) measured from 10 cherry tomatoes stored in OBC (EO)-, OBC (PO)-, PE-sealed and Open containers at 23 ºC for 7 days. The cherry tomatoes stored in the OBC (EO) film reserved the highest overall freshness with a zero moldy rate and a low weight loss.

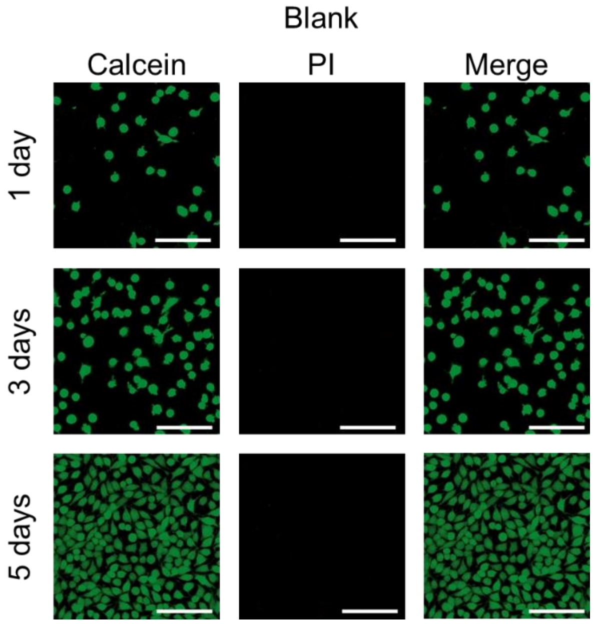

Figure S14. Fluorescence micrographs for growth of L929 cells on a blank sample (the well bottom of a 48-well plate). The number of live cells (green) was increasing over 5 days, indicating no cytotoxicity in the blank sample. Calcein and propidium iodide (PI) marked the live and dead cells, respectively. Scale bars are 100 μm.

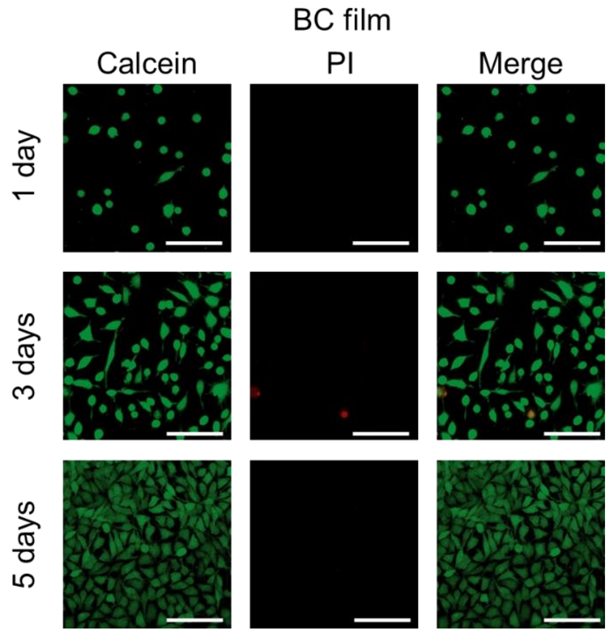

Figure S15. Fluorescence micrographs for growth of L929 cells on a BC film. The number of live cells (green) was increasing over 5 days, indicating the BC film was not cytotoxicity. Calcein and propidium iodide (PI) marked the live and dead cells, respectively. Scale bars are 100 μm.

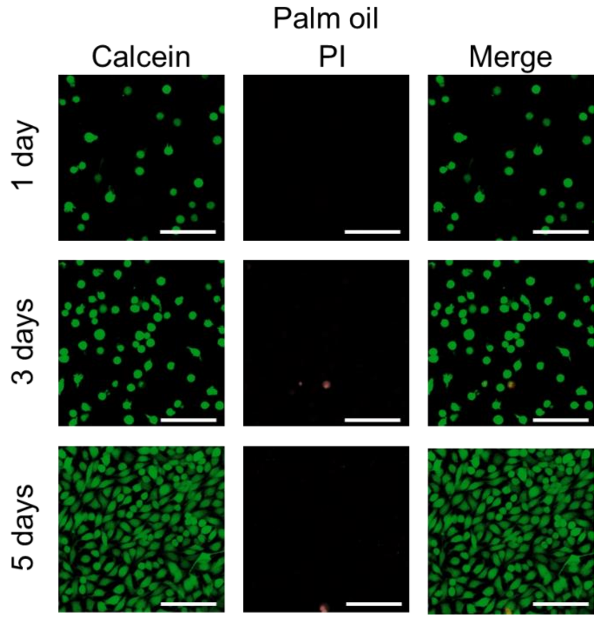

Figure S16. Fluorescence micrographs for growth of L929 cells on palm oil. The number of live cells (green) was increasing over 5 days, indicating that the palm oil was not cytotoxic. Calcein and propidium iodide (PI) marked the live and dead cells, respectively. Scale bars are 100 μm.

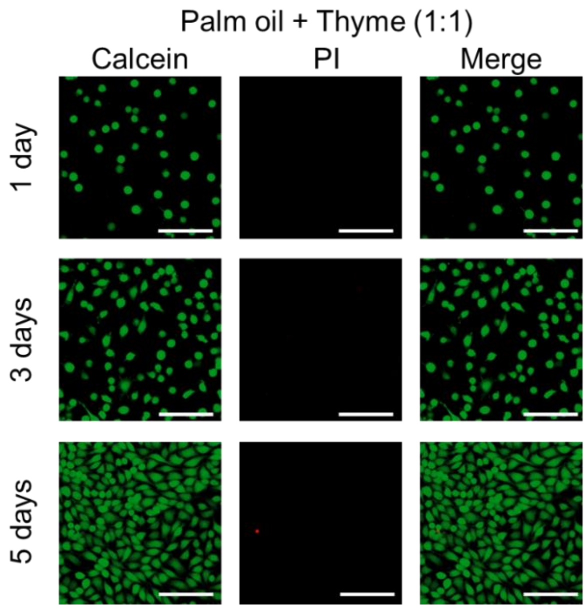

Figure S17. Fluorescence micrographs for growth of L929 cells on palm oil diluted thyme essential oil. The number of live cells (green) was increasing over 5 days, indicating the diluted thyme essential oil was not cytotoxic. Calcein and propidium iodide (PI) marked the live and dead cells, respectively. Scale bars are 100 μm.

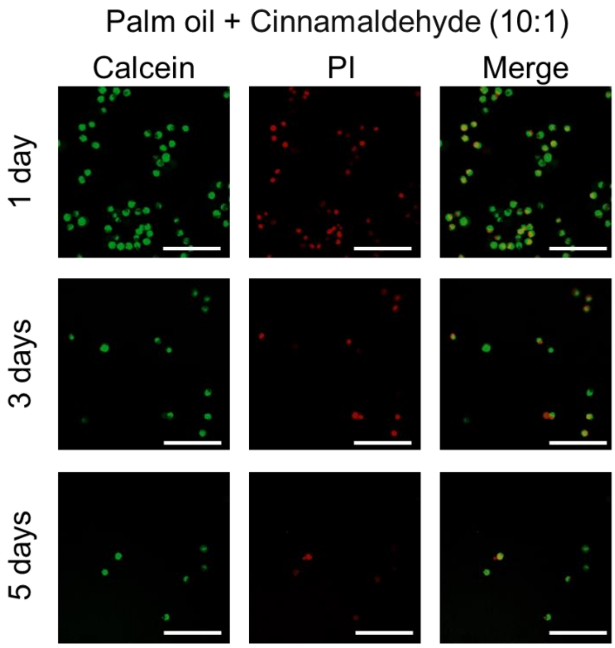

Figure S18. Fluorescence micrographs for growth of L929 cells on palm oil diluted cinnamaldehyde with a volume ratio of 10:1. The number of live cells (green) was decreasing over 5 days, indicating that cinnamaldehyde was cytotoxic. However, cinnamaldehyde is a legal food additive approved by the European Union and U.S. Food and Drug Administration at a recommended level of 1.5 mg/kg since the toxicity of cinnamaldehyde toxicity is concentration-dependent.

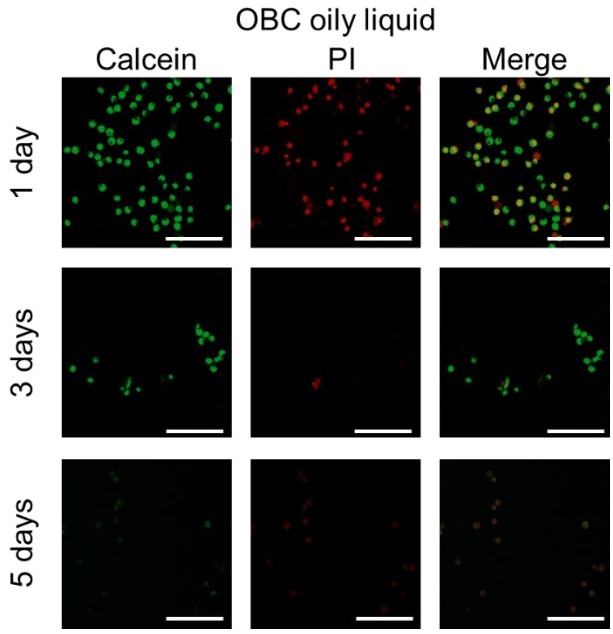

Figure S19. Fluorescence micrographs for growth of L929 cells on the oily liquid of OBC (palm oil: thymol essential oil: cinnamaldehyde = 5:5:1, v/v). The number of live cells (green) was decreasing over 5 days, confirming the cytotoxicity of cinnamaldehyde. However, cinnamaldehyde was not detected in foods stored by OBC film if the food has no physical contact with OBC or the food was washed with water after physical contact (Figure 2g), meaning OBC was safe to use in food storage. Scale bars are 100 μm.

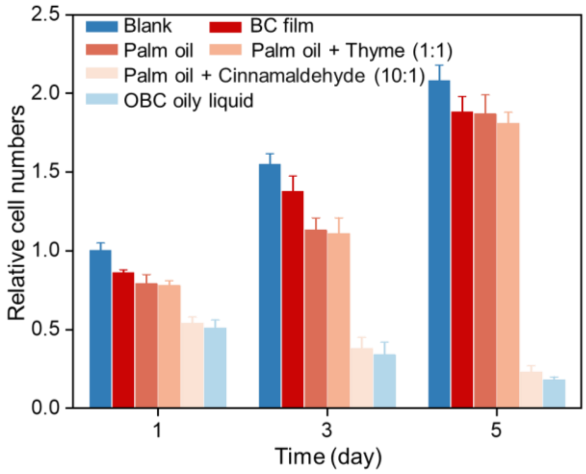

Figure S20. Relative cell numbers of blank, BC film, palm oil, palm oil : thymol essential oil = 1:1, palm oil : cinnamaldehyde = 10:1, and OBC oily liquid. The relative cell numbers in blank, BC, palm oil, and palm oil : thyme essential oil = 1:1 groups was increased over 5 days, indicating that the cells were growing and the samples were not cytotoxic. The relative cell numbers of cinnamaldehyde-containing samples were attenuated, indicating the cells were dying and the samples were cytotoxic. Relative cell numbers data were consistent with the cell growth data shown in Figures S14-S19.

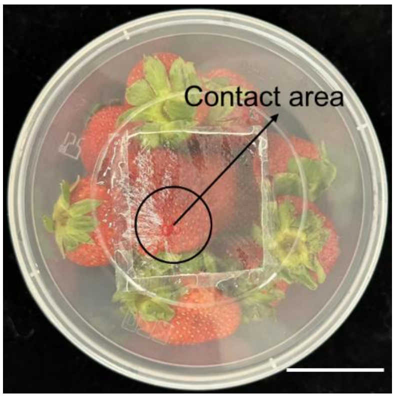

Figure S21. A photograph of strawberries that were contact with OBC film. The compositions of these strawberries before and after being washed with water were measured by GC to reveal whether cinnamaldehyde residues left on the surface (Figure 2g). Scale bar is 3 cm.

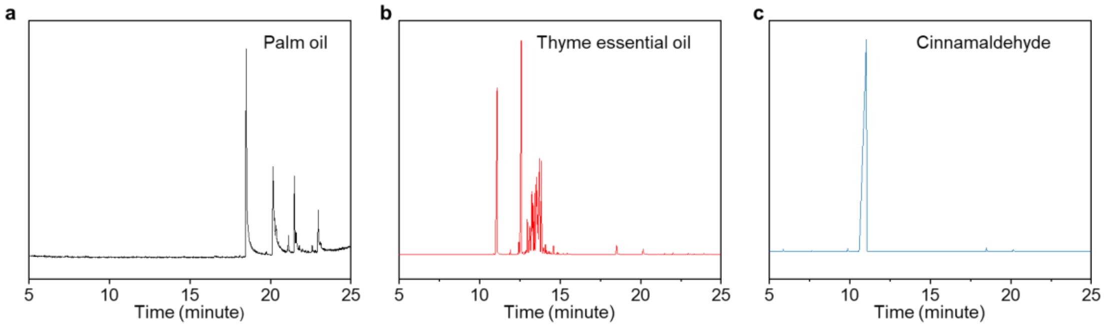

Figure S22. GC spectra of palm oil (a), thyme essential oil (b), and cinnamaldehyde (c).

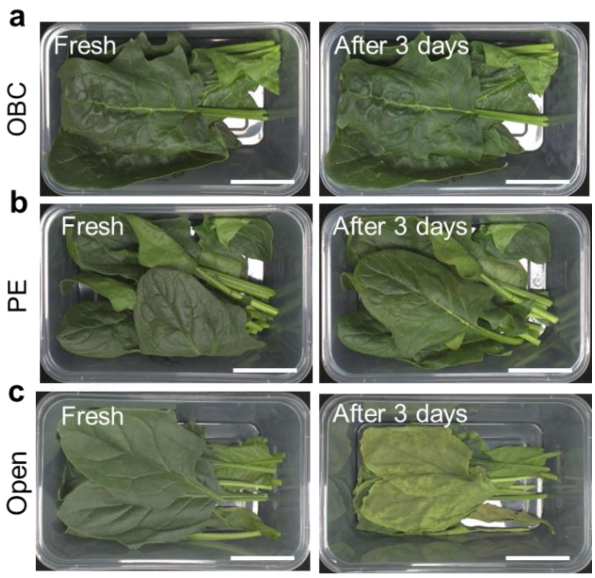

Figure S23. Photographs of spinaches (a kind of leaf vegetable) before and after being stored in OBC-sealed, PE-sealed, and open containers at 23 ºC for 3 days. No change was observed on spinaches stored in the OBC and PE containers. Water loss and drying were apparent on spinaches in the open container. Scale bars are 4 cm.

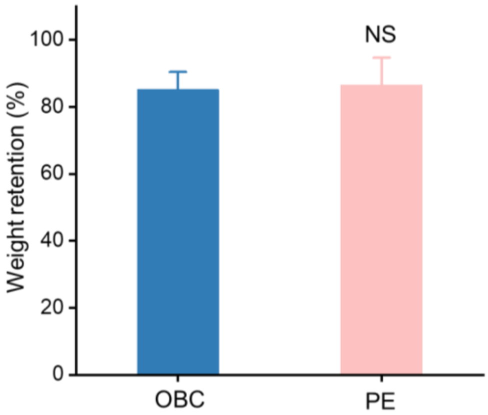

Figure S24. Weight retention of 3 lettuce samples stored in OBC and PE conditions at 23 °C for 3 days. The residual weight of the OBC group lettuce was 85.2% compared to 86.5% of the PE group lettuce. NS, the data between these two groups were not statistically significant, showing that OBC and PE have similar freshness retention properties for lettuce.

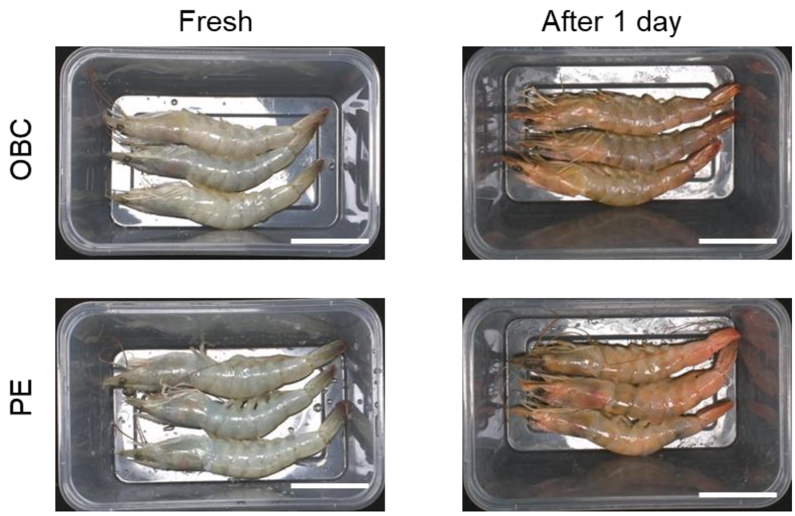

Figure S25. Photographs of shrimps before and after being stored in OBC-sealed, and PE-sealed containers at 23 ºC for 1 day. Shrimps stored by OBC presented reduced oxidative blackening and astaxanthin reddening compared to that stored by PE. Scale bars are 4 cm.

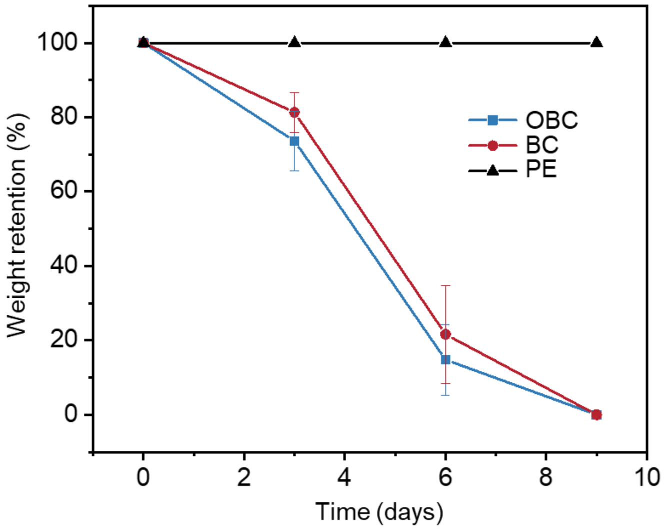

Figure S26. Mass variation of OBC, BC, and PE films as a function of degradation time under soil, showing a similar rate of degradation for BC and OBC. PE showed no mass loss for 9 days, which suggests no degradation occurred.

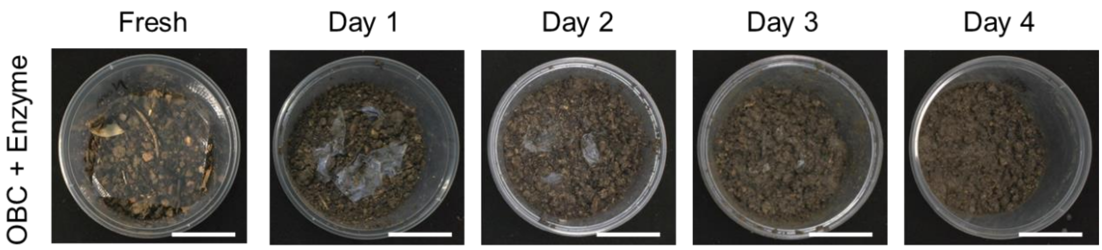

Figure S27. Photographs of an OBC film that gradually degraded in moist soil containing cellulase at 30 ºC. Adding cellulase enzyme to soil accelerated the degradation rate of OBC film. Scale bars are 4 cm.

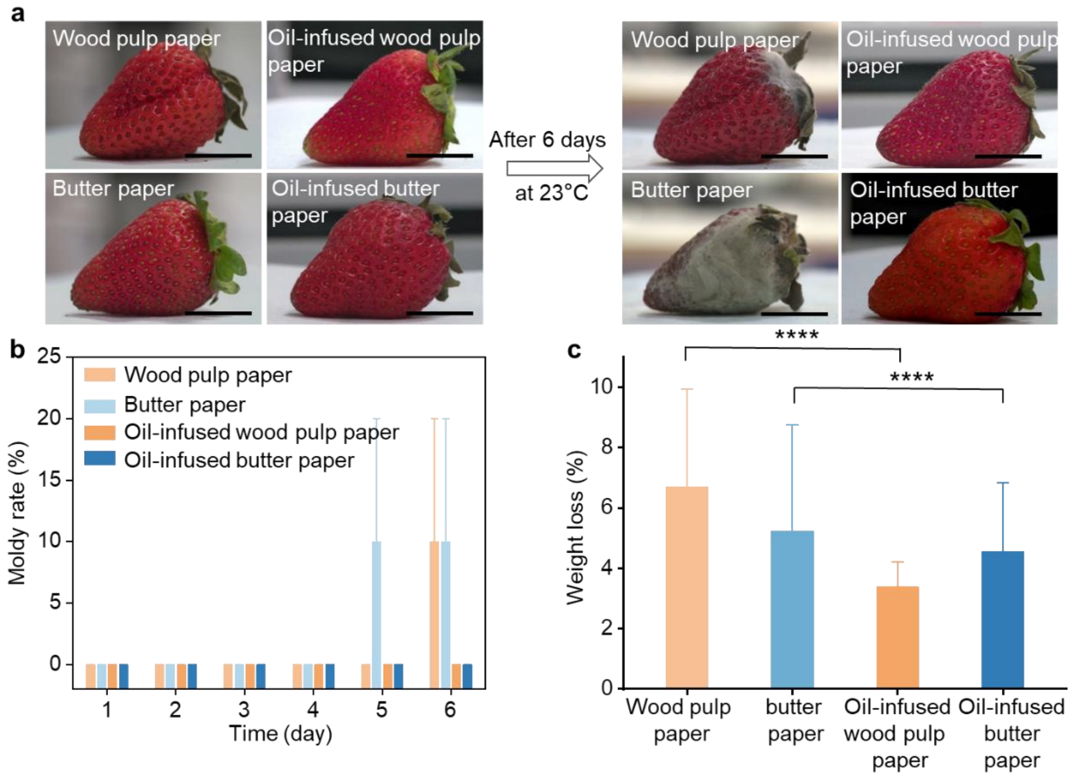

Figure S28. Fruit storage performance of oil-infused wood pulp paper and oil-infused butter paper. (a) Photographs comparing the appearance changes of strawberries before and after being stored in wood pulp paper-sealed, oil-infused wood pulp paper-sealed, butter paper-sealed, and oil-infused butter paper-sealed containers at 23 ºC for 6 days. Scale bars were 2 cm. (b,c) moldy rate (b), and weight loss (c) from 10 strawberries stored in the 4 kinds of papers at 23 ºC for 6 days. ****p < 0.0001 by two-way ANOVA, compared with the response of the control group. These results verify the broad applicability of the oil infusion strategy on paper-based packaging materials.

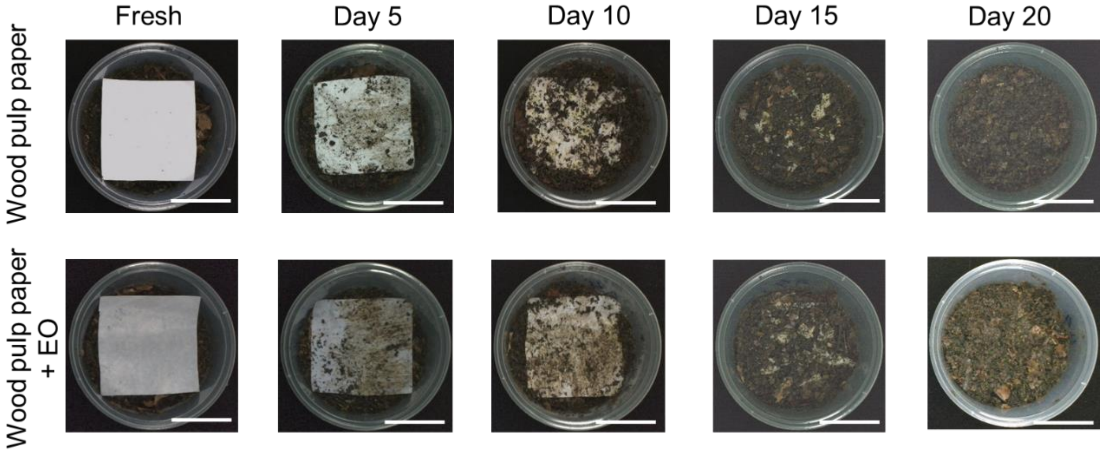

Figure S29. Photographs of wood pulp paper and oil-infused wood pulp paper (wood pulp paper + essential (EO)) in moist soil containing cellulase at 30 ºC, showing a comparable degradation rate. The complete degradation of wood pulp paper took longer time (20 days) compared that of bacterial cellulose (4 days), due to their intrinsic structural difference. Scale bars are 4 cm.

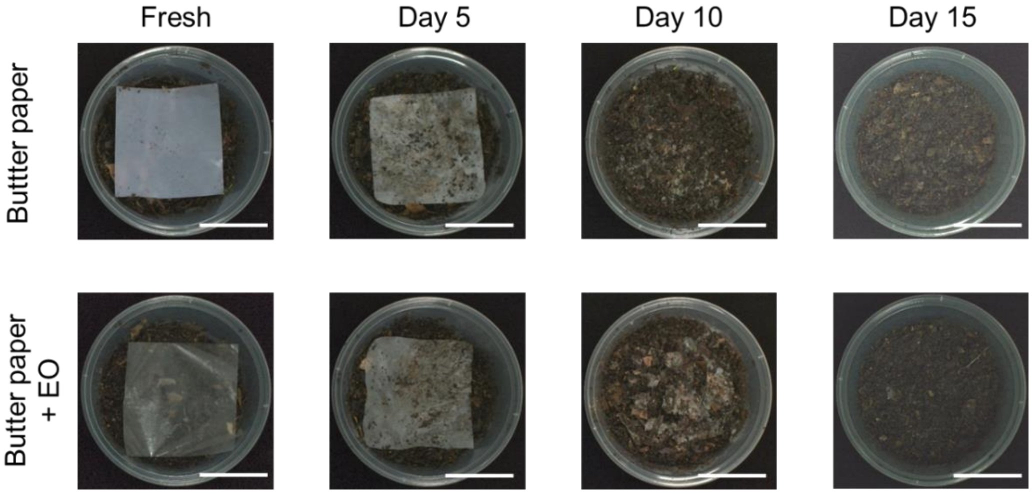

Figure S30. Photographs of butter paper and oil-infused butter paper (butter paper + EO) in moist soil containing cellulase at 30 ºC, showing a comparable degradation rate. Scale bars are 4 cm.
